# Differential protein expression and enriched pathways in pediatric sepsis: identification of novel brain-associated biomarkers revealed through proteomic profiling

**DOI:** 10.1186/s10020-025-01397-x

**Published:** 2025-11-26

**Authors:** Vincenzo Stranges, David Tweddell, Enis Cela, Maria Morello, Mark Daley, Gediminas Cepinskas, Douglas D. Fraser

**Affiliations:** 1 Maternal and Child Health and Urological Sciences, Policlinico Umberto I, Rome, Italy; 2 Computer Science, Western University, London, ON Canada; 3 Physiology and Pharmacology, Western University, London, ON Canada; 4 Experimental Medicine, University of Rome Tor Vergata, Rome, Italy; 5 Epidemiology and Biostatistics, Western University, London, ON Canada; 6 Medical Biophysics, Western University, London, ON Canada; 7 Anatomy and Cell Biology, Western University, London, ON Canada; 8 London Health Sciences Centre Research Institute, 800 Commissioners Road East, London, ON Canada; 9 Pediatrics, Western University, London, ON Canada; 10 Clinical Neurological Sciences, Western University, London, ON Canada; 11 Children’s Health Research Institute, London, ON Canada; 12 GSK Chair in Clinical Pharmacology, Western University, London, ON Canada

**Keywords:** Pediatrics, Sepsis, Proteomics, Biomarkers, Signaling pathway, Brain injury

## Abstract

**Background:**

Sepsis, defined by confirmed or suspected infection with systemic inflammatory response syndrome, requires robust biomarker identification. Proteomics enables protein quantification and expression analysis across disease states. This study investigated differential protein expression patterns, particularly brain-associated proteins, between sepsis patients and healthy controls, while evaluating temporal variations and relevant molecular pathways.

**Methods:**

Study participants were prospectively enrolled based on established pediatric sepsis criteria with clinical and blood samples collected. Plasma protein concentrations were quantified using Nucleic Acid Linked Immuno-Sandwich Assay methodology. Statistical analyses incorporated conventional statistics, bioinformatics and machine learning approaches.

**Results:**

The study cohorts comprised 23 age- and sex-matched participants: pediatric sepsis patients (median 11 years, IQR 9.5–14) and healthy controls (median 11 years, IQR 7.8–13; *P* = 0.809). Analyses revealed 59 differentially expressed proteins (DEPs) on Pediatric Intensive Care Unit Day 1 (PICU D1). Random Forest Classification (RFC) with Boruta feature selection identified 29 proteins that facilitated distinct group stratification. Comparison between PICU D1 and D3 samples yielded 34 DEPs, with RFC and Boruta feature selection isolating 9 discriminatory proteins. Multiple proteins were correlated with PELOD-2 scores and mortality (*P* < 0.05). Novel brain-associated proteins demonstrated significant differential expression patterns between PICU D1 and healthy controls, and over 3 days of PICU stay in sepsis patients. PICU D1 samples demonstrated significant pathway upregulation when compared to healthy controls, including “Signaling by Interleukins”, “Cytokine Signaling in Immune system”, and “Interleukin-10 signaling”. By PICU D3, pathways associated with “Generic Transcription Pathway”, “RNA Polymerase II Transcription”, and “Gene expression (Transcription)” exhibited significant downregulation. Protein-protein interaction network analysis revealed TNF and IL1B as critical bridging proteins linking inflammatory and neurological processes. Disease enrichment analysis demonstrated significant over-representation of respiratory pathology-associated genes, with respiratory failure and adult respiratory distress syndrome as the most enriched categories.

**Conclusions:**

Our investigation revealed distinct proteomic signatures in inflammatory and transcriptional pathways, including brain-associated processes, that differentiated pediatric sepsis patients from healthy control participants and exhibited temporal dynamics. The identification of TNF and IL1*B* as bridging proteins between systemic inflammation and neurological processes, combined with respiratory-centric disease enrichment patterns, provides mechanistic insights into sepsis pathophysiology. These alterations may provide insight into the mechanisms underlying sepsis-associated encephalopathy and lingering cognitive impairment in sepsis survivors, warranting further investigation in future studies. The identified molecular signatures present potential diagnostic and prognostic biomarkers for pediatric sepsis management.

**Supplementary Information:**

The online version contains supplementary material available at 10.1186/s10020-025-01397-x.

## Introduction

Sepsis is a life-threatening condition characterized by a suspected or confirmed infection in a patient exhibiting systemic inflammatory response syndrome (SIRS) (Schlapbach et al. 2024). In pediatric populations, sepsis poses a significant risk, particularly in the early years of life, where children are more vulnerable to developing this condition compared to adults (Souza et al. 2022). Additionally, the risk of sepsis is markedly higher in settings with limited socioeconomic resources, which may exacerbate the severity and outcomes of the disease. Pediatric sepsis differs from its adult counterpart in several important ways, including distinct age-based thresholds for vital sign parameters, variations in the immune response, differing comorbidities, and unique epidemiological patterns and outcomes (Watson et al. 2003; de Souza et al. 2021; Schlapbach et al. 2015).

Sepsis involves complex interactions between the immune system and microbial pathogens, profoundly affecting patient outcomes. Infected microorganisms release pathogen-associated molecular patterns that trigger immune responses, leading to the expression of cytokines, chemokines, and acute-phase proteins (Kumar et al. 2013; Heckenberg et al. 2014; Mook-Kanamori et al. 2011; Iwasaki and Medzhitov 2010). Host cell damage also releases damage-associated molecular patterns, amplifying the inflammatory response (Wiersinga et al. 2014; Lu et al. 2014). This cascade can progress to a cytokine storm, characterized by excessive production of pro-inflammatory mediators including TNF-α (Tumor Necrosis Factor-α), IL (interleukin)−1β, and IL-6, with TNF-α concentrations in sepsis patients approximately 10-fold higher than healthy individuals and directly correlating with mortality (Shakoory et al. 2016). The cytokine storm disrupts the blood-brain barrier (BBB) through endothelial dysfunction and matrix metalloproteinase activation, enabling pro-inflammatory mediators to flood the brain and activate microglia, ultimately triggering neuroinflammation (Xin et al. 2023; Daniels et al. 2014).

A compromised BBB leads to heightened permeability and facilitates the entry of immune cells into the central nervous system, potentially contributing to neuroinflammation and altered neurological function (Barichello et al. 2022; Annane and Sharshar 2015) Sepsis-induced acute brain dysfunction, often referred to as sepsis-associated encephalopathy (SAE), is characterized by altered mental status, delirium, cognitive impairment, and in severe cases, coma (Dumbuya et al. 2023). The clinical significance of studying cytokines and brain-related proteins lies in their potential as biomarkers for early detection and monitoring of these life-threatening complications, as cytokine levels correlate with disease severity and patient outcomes (Chaudhry et al. 2013).

Proteomics, the study of proteins, their post-translational modifications, and their functional roles, offers valuable insights into the molecular mechanisms underlying diseases such as sepsis (Petricoin et al. 2002; Miao et al. 2021). In the context of sepsis, proteomic analysis can be instrumental in identifying potential biomarkers, assessing their differential expression across disease states, and tracking changes over the course of hospitalization (Lim et al. 2023). By comparing the protein profiles of sepsis patients to those of healthy individuals, researchers can uncover critical insights into the pathophysiology of sepsis and its progression (Leonard et al. 2024).

This case-control study aimed to: (1) identify molecular pathways with significant alterations in protein expression on PICU (Pediatric Intensive Care Unit) Day 1 (D1) and Day 3 (D3); (2) examine associations between these protein expression changes and clinical variables, including disease severity, treatments, and patient outcomes; and (3) identify novel brain-associated proteins that may play a role in the progression of sepsis in critically ill children.

## Methods

This study was approved by the Western University, Human Research Ethics Board. Our experimental methods were performed in accordance with ethical standards of the responsible committee on human experimentation and with the Helsinki Declaration of 1975. Informed consent was obtained from the legal guardians of all pediatric patients admitted to the PICU with sepsis, and both guardian consent and assent were secured for healthy control participants.

### Sepsis criteria

Pediatric sepsis patients in the PICU, aged 1 month to 17 years of age, were enrolled based on established clinical criteria. There were no exclusion criteria. Initially, we used the Systemic Inflammatory Response Syndrome (SIRS) criteria, which include fever, tachycardia, tachypnea, and/or abnormal white blood cell (WBC) count, as an initial screening tool for sepsis. We then applied the International Consensus Criteria for Pediatric Sepsis and Septic Shock criteria (Schlapbach et al. 2024; Goldstein et al. 2005), which account for signs of organ dysfunction. These criteria include hypotension (age-specific low blood pressure), altered mental status (e.g., lethargy or confusion), oliguria (reduced urine output), poor peripheral perfusion (such as cold extremities or delayed capillary refill), and/or elevated lactate levels or abnormal blood gases, indicative of impaired perfusion. Confirmed sepsis was defined as the presence of clear evidence of an infection, including positive microbiological cultures and/or viral testing (antigen or polymerase chain reaction), imaging findings showing an infection (e.g., pneumonia, abscess), or clinical signs suggestive of infection (e.g., fever, elevated white blood cell count). In contrast, suspected sepsis refers to cases where definitive microbiological evidence is lacking, or when clinical signs of sepsis are present but confirmation through positive cultures or other diagnostic tests is still pending.

### Data collection and blood sampling

Patient characteristics included age, sex, pathogen, source of infection, chest x-ray, mechanical ventilation, hemodynamic support, number of PICU days, and total number of hospital days. Comorbidities were included with a focus on organ-specific conditions, categorizing each patient’s health status based on systems affected by the disease. Illness severity scores were calculated, including Pediatric Risk of Mortality III (PRISM III) score, Pediatric Index of Mortality 2 (PIM 2) score, daily Pediatric Logistic Organ Dysfunction 2 (PELOD-2) score, and Glasgow coma scale (GCS). PICU D1 was defined as the first 24 h of the patient’s admission to the PICU, starting from the time of admission. Subsequent days were counted consecutively, with D3 being the third full calendar day after admission.

Study blood was obtained at the time of clinical draws, using citrate vacutainer blood collection tubes. To obtain the plasma, blood was centrifuged at 1500 × g for 15 min at 4 °C. The plasma was isolated, aliquoted, and stored frozen at −80 °C. Freeze and thaw cycles were avoided. For comparison with sepsis patients, age- and sex-matched healthy control subjects without disease or acute illness were identified from the Translational Research Centre, London, Ontario (www.translationalresearch.ca) (Brisson et al. 2012; Gillio-Meina et al. 2013).

### Nucleic Acid Linked Immuno-Sandwich Assay (NULISA)

Protein expression was measured in pediatric plasma samples using the Nucleic Acid Linked Immuno-Sandwich Assay (NULISA), enabling comparison across cohorts and the assessment of correlations between identified pathways and clinical variables. Plasma samples were thawed and centrifuged at 10,000 g for 10 min. Exactly 10 µL of supernatant from the sample was added to 96-well plates and assayed with the NULISAseq CNS Panel 120, targeting mostly neurodegenerative disease-related protein markers and inflammation and immune response-related cytokines and chemokines. Pair of antibodies for each target, (a) a capture antibody conjugated with partially double-stranded DNA containing a poly-A tail and a target-specific molecular identifier (TMI) and (b) a detection antibody conjugated with another partially double-stranded DNA containing a biotin group and a matching target-specific barcode (TMI) was pooled into a multiplex panel. Incubation of the antibodies with a sample containing the target antigen resulted in the formation of an immunocomplex. The formed immunocomplexes were subsequently captured by addition of paramagnetic oligo-dT beads via dT-polyA hybridization and the sample matrix and unbound detection antibodies were removed by washing. As dT-polyA binding was sensitive to salt concentration, the immunocomplexes were then released into a low-salt buffer. After removal of the dT beads, a second set of paramagnetic beads coated with streptavidin was introduced for a second capture of the immunocomplexes in the solid phase. Subsequent washes removed any unbound capture antibodies leaving only intact immunocomplexes on the beads. Then, a ligation reaction mix containing T4 DNA ligase and a specific DNA ligator sequence along with a sample-specific molecular identifier (SMI) was added, allowing the ligation of the proximal ends of the DNA attached to the paired antibodies and, thus, generating a DNA reporter molecule containing unique pairs of TMIs and a SMI. The amount of the DNA reporter was then quantified by next-generation sequencing with an Illumina NextSeq 2000 system.

### Statistical analysis

Non-parametric methods were used for all comparisons. Demographic and cohort data were summarized using median (interquartile range [IQR]) for continuous variables and frequency (%) for categorical variables. Comparisons of categorical variables were performed using a chi-square test, and continuous variables were compared using Mann-Whitney U or Kruskal-Wallis tests, depending on the number of groups. All statistical analyses were conducted using GraphPad Prism (Version 8.4.0; GraphPad Software, San Diego, California, USA).

### Clinical Outcome-Protein associations

Associations between protein expression levels and PELOD-2 scores were assessed using Pearson correlation coefficients. PELOD-2 scores were treated as continuous variables for this analysis. Correlations were calculated independently for each time point, comparing D1 protein expression with D1 PELOD-2 scores and D3 protein expression with D3 PELOD-2 scores. Both raw p-values and those adjusted for multiple comparisons using the Benjamini-Hochberg FDR procedure, are shown.

Relationships between protein expression and mortality were evaluated using point-biserial correlations, which are appropriate for associations between continuous variables (protein expression) and dichotomous outcomes (mortality status: 0 = alive, 1 = deceased). Correlations were calculated for protein expression levels at both D1 and D3 time points against the final mortality outcome. Both raw p-values and those adjusted for multiple comparisons using the Benjamini-Hochberg FDR procedure, are shown.

### Machine learning models: protein feature selection for sepsis classification

Decision tree methods, when assembled into ensembles, are useful for determining important features that predict class membership (and binary classification tasks) when working with high dimensional data. Normalized protein expression data was processed to identify key features that differentiate between cohorts (Sepsis PICU D1 vs. HC [Healthy Control]) and timepoints (Sepsis PICU D1 vs. Sepsis PICU D3). A random forest classifier (RFC) (Breiman 2001) comprising 5,000 decision trees (maximum depth of 5 leaves) was trained for each of these classification tasks. The Boruta feature selection method (Kursa and Rudnicki 2010) was subsequently applied to the trained classifiers. In each case, a second random forest model (5,000 estimators, maximum depth of 5) was then trained using only the selected features to assess the relative importance of each metabolite in the classifier. The important features, and the resulting capability of the RFC is illustrated using t-stochastic neighbor embedding (t-SNE) (Van der Maaten and Hinton 2008) for dimensionality reduction and visualization. The machine learning models were implemented using python 3.11.0, pandas 2.2.3, numpy 2.2.5, scikit-learn 1.6.1, and boruta_py 0.3.0.

### Computational bioinformatics

Protein expression comparisons were conducted using empirical Bayes moderated t-tests with the limma R package. To account for multiple testing, p-values were adjusted using the false discovery rate (FDR). A positive log₂ (fold change) indicated up-regulation in one group relative to another, while a negative value indicated down-regulation. Proteins exhibiting a significance level of adjusted p≤ 0.05 and a fold change ≥ 2 were mapped to Entrez gene identifiers and further analyzed using Reactome term over-representation analysis (ORA; https://reactome.org/).

### Clinical Variable-Pathway associations

We employed a graph network approach to analyze pathway associations with clinical observations (Spagnolo et al. 2025). The pathways and clinical variables are represented as different types of nodes in a bipartite graph, where edges indicate the degree of association between nodes. To evaluate a pathway’s association with a clinical variable, we used the set of implicated proteins obtained from Reactome over-representation analysis (ORA). For each protein, we assessed two features: the correlation with the clinical variable (using Pearson’s correlation coefficient ρ) and the protein’s relative importance R in differential expression relative to a comparator group. Inspired by the volcano plot, which depicts impact (log2 fold change) and significance (-log10(adjusted p-value), we combined these features into a single measure of relative importance:

$$\:\mathrm{R}\:=\:\sqrt{\left({\mathrm{log}}_2\left(\text{fold change}\right)\right)^2+\left(-\log_{10}\left(\text{adjusted p-value}\right)\right)^2}$$

For each protein, the weight *w* for its association with a clinical variable is calculated as:

$$\it \mathrm w=\mathrm\rho\:\mathrm R$$

If the Pearson’s ρ was significant for a protein/clinical variable pair (p< 0.05), an edge was added to the graph with weight *w*, indicating a connection between the pathway (via the protein) and the clinical variable.

### Protein-Protein interaction and disease enrichment

Protein-protein interactions were analyzed using STRING (https://string-db.org/) to identify functional networks among differentially expressed proteins. The 59 differentially expressed proteins, ranked by FDR p-value, were uploaded to the STRING database. Analysis parameters were configured as follows: full STRING network selection, high confidence score threshold (0.7), and the first shell set to 1 with no second-shell interactions included. Protein clustering was performed using k-means clustering with k = 4 to identify optimal functional clusters within the interaction network.

Disease-enrichment analysis was performed using the built-in enrichment functionality of STRING. The analysis utilized the DISEASES database, which integrates disease-gene associations from automated text mining, manually curated literature, cancer mutation data, and genome-wide association studies. Enrichment was assessed against a whole genome background to identify diseases significantly overrepresented among the input protein set. The statistical significance of disease enrichment was determined using a hypergeometric test with Benjamini-Hochberg FDR correction for multiple testing. Diseases with FDR-adjusted p< 0.05 were considered significantly enriched.

## Results

### Patient demographic and clinical data

**Fig. 1:**
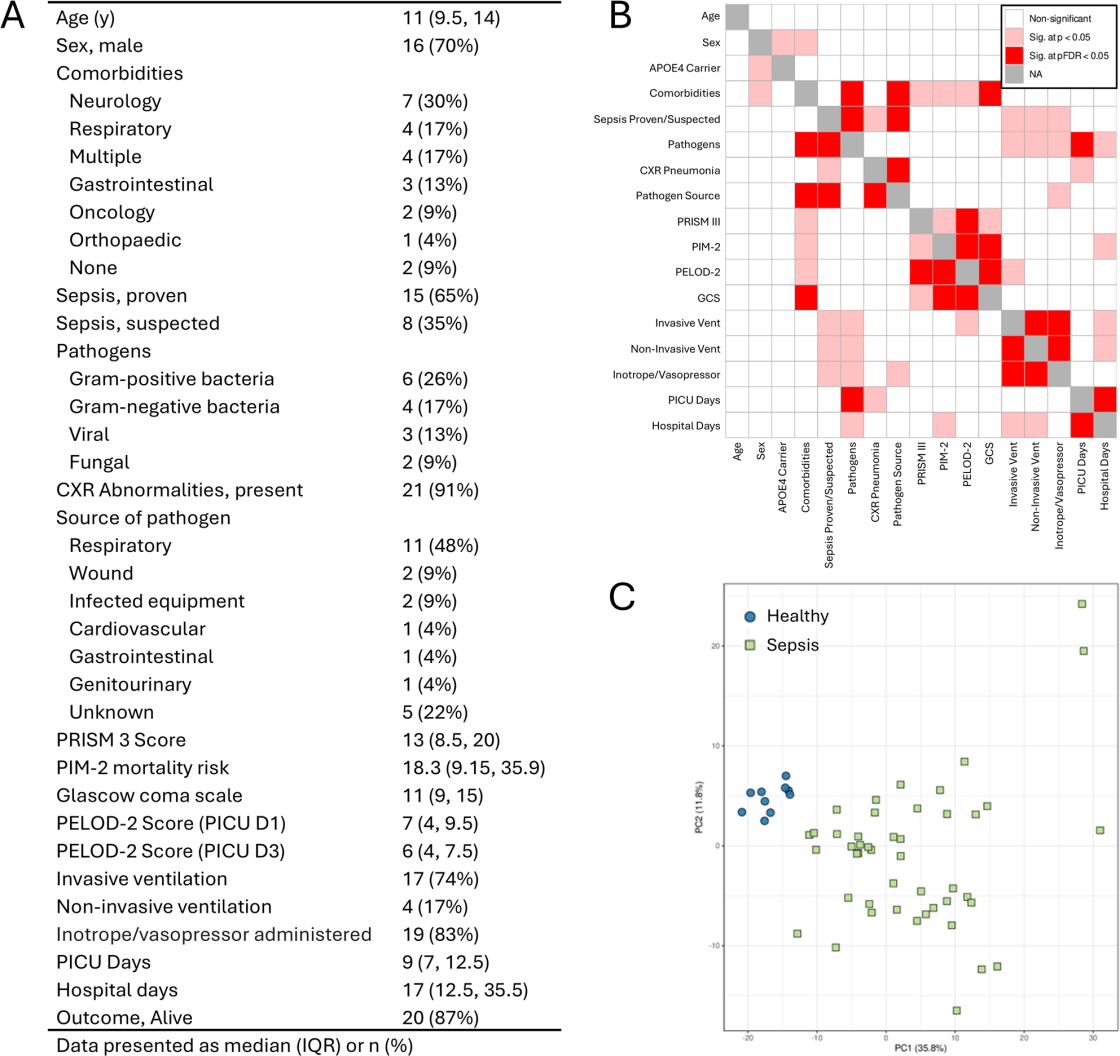
Patient variables with correlations and grouping of samples. **A** Patient demographics and biochemistry. **B** Discrete heatmap showing associations between all factors in the study. The strength of the associations is defined by the color of the blocks as non-significant (white), significant at p<0.05 (pink), significant at FDR < 0.05 (red). **C** Scatterplots of the first two principal components from the normalized dataset. Colors represent the different cohorts in the dataset. Samples cluster according to one or more experimental factors to reveal the underlying cohorts

The study cohorts comprised 23 age- and sex-matched participants: pediatric sepsis patients (median 11 years, IQR 9.5–14) and healthy controls (median 11 years, IQR 7.8–13; *p* = 0.809). Demographic and clinical data for the sepsis patients are shown in Fig. 1A. Of these, *n* = 15 (65%) had confirmed sepsis, while *n* = 8 (35%) were classified as suspected sepsis. Most patients had comorbidities (*n* = 21, 91%), with *n* = 7 (30%) having neurological conditions. Bacterial infections were the most prevalent (*n* = 10, 43%), and most infections were respiratory (*n* = 11, 48%), consistent with chest X-ray abnormalities in *n* = 21 (91%) of patients. The median GCS score on admission was 11 (IQR 9–15). The PRISM III score had a median of 13 (IQR 8.5–20). A significant portion of patients required invasive mechanical ventilation (17, 74%) and pharmacological circulatory support (*n* = 19, 83%). The median PICU stay was 9 days (IQR 7–12.5), and the median total hospital stay was 17 days (IQR 12.5–35.5). Associations between variables are summarized in Fig. 1B. A scatterplot showed that healthy controls clustered tightly, while septic patients exhibited greater heterogeneity (Fig. 1C).

### Differentially expressed proteins

**Fig. 2:**
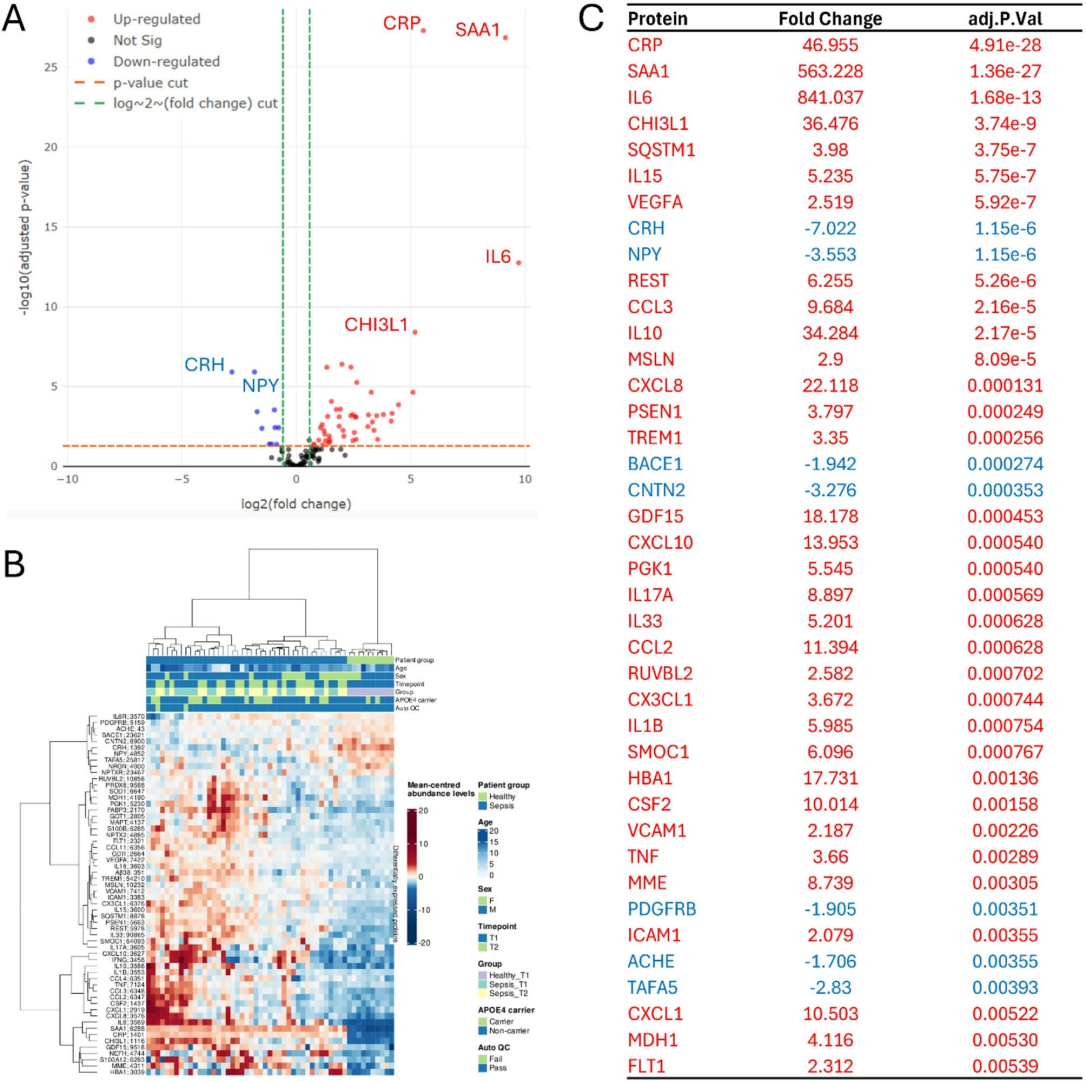
Association tests between sepsis PICU D1 patients and healthy controls shown graphically and with a ranking of significant differentially expressed proteins. **A** Volcano plot depicting significance versus magnitude of protein expression changes. The x-axis represents the log2 fold change, while the y-axis shows -log10 transformed p-values. Proteins with significant differences between samples are colored: red for up-regulated and blue for down-regulated. For performance reasons, in cases with large datasets, the non-significant proteins (black) are shown as a representative sub-sample. Vertical green and horizontal orange lines denote the thresholds for fold change and p-value, respectively. **B** Heatmap illustrating protein intensity levels per sample, normalized to the average intensity across all samples. Proteins are listed along the Y-axis, and samples are along the X-axis. The colour scale indicates protein abundance: red for higher levels and blue for lower levels relative to the average. The heatmap displays up to 1000 proteins and 1000 samples, with features and samples selected randomly if the dataset exceeds these limits. **C** A list of the 40 leading differentially expressed proteins. Proteins upregulated in sepsis D1 patients are marked in red, while downregulated proteins are marked in blue. This list focuses on proteins exhibiting significant changes in abundance, based on adj P<0.05

Differentially expressed proteins (DEPs) between sepsis PICU D1 patients and healthy controls are displayed in a Volcano plot (Fig. 2A), with positive values indicating upregulation and negative values indicating downregulation. A heatmap (Fig. 2B) shows normalized protein intensity levels per sample. A total of 59 DEPs were identified. Out of the top 40 DEPs (Fig. 2C), 33 were significantly upregulated and 7 were downregulated (adjusted p< 0.05). Notable upregulated proteins on PICU D1 included C-Reactive protein (CRP; FC 46.955; adj. p-value 4.91e-28), Serum Amyloid A-1 (SAA1; FC 563.228; adj. p-value 1.36e-27), IL-6 (FC 841.037; adj. p-value 1.68e-13), and Chitinase-3-like protein 1 (CHI3L1; FC 36.476; adj. p-value 3.74e-09), while Corticotropin Releasing Hormone (CRH; FC −7.022; adj. p-value 1.15e-06) and Neuropeptide Y (NPY; FC -3.553; adj. p-value 1.15e-06) were significantly downregulated (Fig. 2C). The RFC, with Boruta feature selection, identified 29 protein features that effectively separated the groups into distinct, tightly clustered categories (Supplementary Fig. 1). Associations between DEPs and clinical variables are shown in Supplementary Fig. 2, with box colors representing correlation coefficients (p< 0.05).

**Fig. 3:**
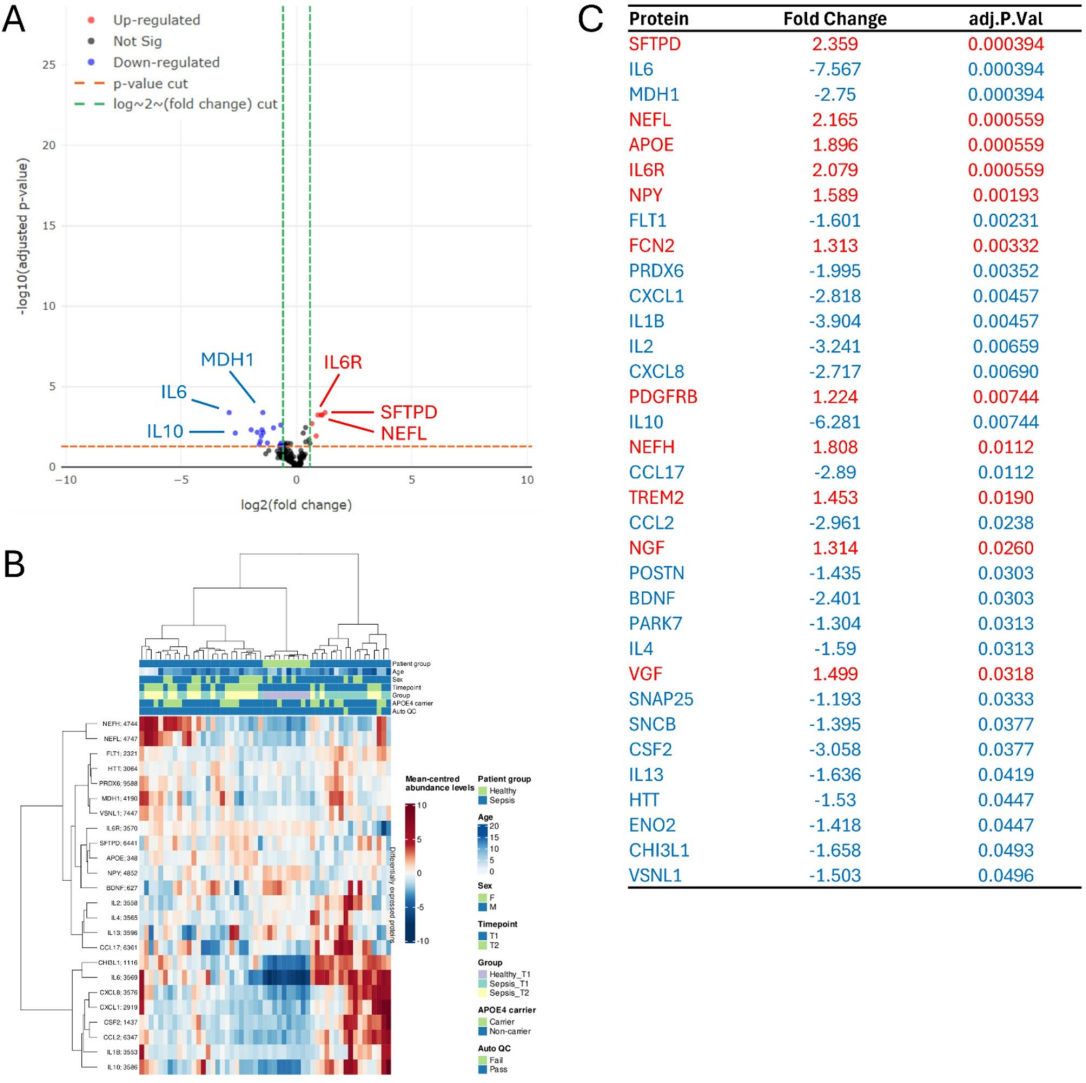
Association tests between sepsis PICU D1 and sepsis PICU D3 patients shown graphically and with a ranking of significant differentially expressed proteins. **A** Volcano plot depicting significance versus magnitude of protein expression changes. The x-axis represents the log2 fold change, while the y-axis shows -log10 transformed p-values. Proteins with significant differences between samples are colored: red for up-regulated and blue for down-regulated. For performance reasons, in cases with large datasets, the non-significant proteins (black) are shown as a representative sub-sample. Vertical green and horizontal orange lines denote the thresholds for fold change and p-value, respectively. **B** Heatmap illustrating protein intensity levels per sample, normalized to the average intensity across all samples. Proteins are listed along the Y-axis, and samples are along the X-axis. The colour scale indicates protein abundance: red for higher levels and blue for lower levels relative to the average. The heatmap displays up to 1000 proteins and 1000 samples, with features and samples selected randomly if the dataset exceeds these limits. **C** A list of all differentially expressed proteins. Proteins upregulated in sepsis D1 patients are marked in red, while downregulated proteins are marked in blue. This list focuses on proteins exhibiting significant changes in abundance, based on adj P<0.05

DEPs between PICU D1 and D3 patients are shown in the Volcano plot, with positive values indicating upregulation and negative values indicating downregulation (Fig. 3A). A heatmap (Fig. 3B) displays protein intensity levels per sample, normalized to the average intensity across samples. A total of 34 DEPs were identified, with 11 significantly upregulated and 23 significantly downregulated. Notably upregulated proteins included on PICU D3: Surfactant Protein D (SFTPD; FC 2.359; adj. p-value 3.94e-04), Neurofilament Light Chain (NEFL; FC 2.165; adj. p-value 5.59e-04), Apolipoprotein A (APOE; FC 1.896; adj. p-value 5.59e-04), and interleukin-6 receptor (IL6R; FC 2.079; adj. p-value 5.59e-04), while IL6 (FC -7.567; adj. p-value 3.94e-04) and Malate Dehydrogenase 1 (MDH1; FC -2.750; adj. p-value 3.94e-04) were significantly downregulated (Fig. 3C). Associations between DEPs and clinical variables are shown in Supplementary Fig. 3, with boxes colored by correlation coefficient values (p< 0.05). The RFC, combined with Boruta feature selection, identified 9 protein features that somewhat separated the classes (Supplementary Fig. 1C). Associations between DEPs and clinical variables are shown in Supplementary Fig. 3, with box colors representing correlation coefficients (p< 0.05).

**Fig. 4:**
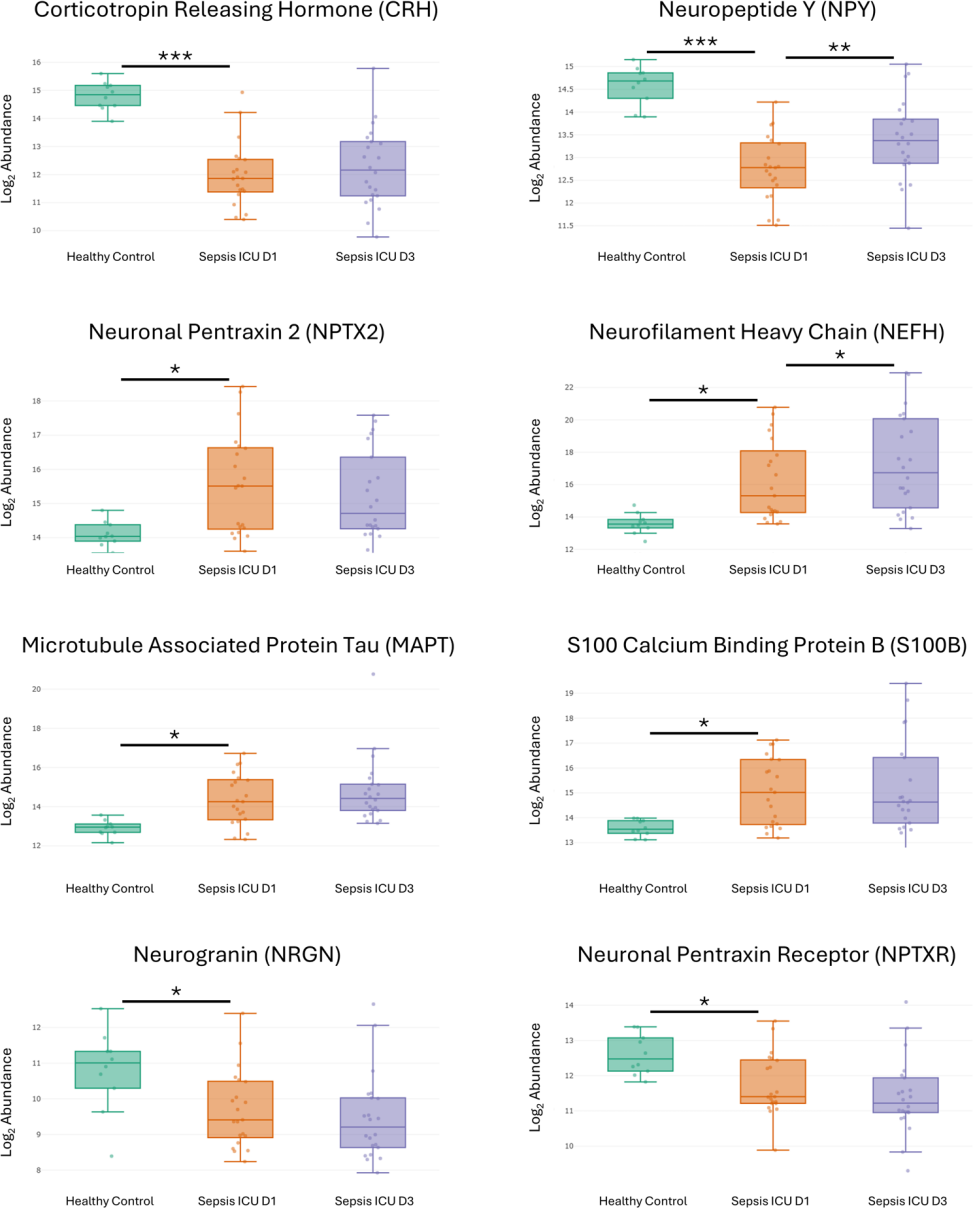
Box Plots illustrating the log2 abundance levels of eight different brain-associated proteins across three groups: Healthy Control, Sepsis PICU D1, and Sepsis PICU D3. The proteins analyzed include Corticotropin Releasing Hormone (CRH), Neuropeptide Y (NPY), Neuronal Pentraxin 2 (NPTX2), Neurofilament Heavy Chain (NEFH), Microtubule Associated Protein Tau (MAPT), S100 Calcium Binding Protein B (S100B), Neurogranin (NRGN), and Neuronal Pentraxin Receptor (NPTXR). The asterisk indicates the level of significance: 1 asterisk indicates a p<0.05, 2 asterisks a p<0.005, 3 asterisks a p<0.001. The horizontal line below the asterisks indicates the groups that have a significant difference in terms of median in the expression of proteins

**Fig. 5:**
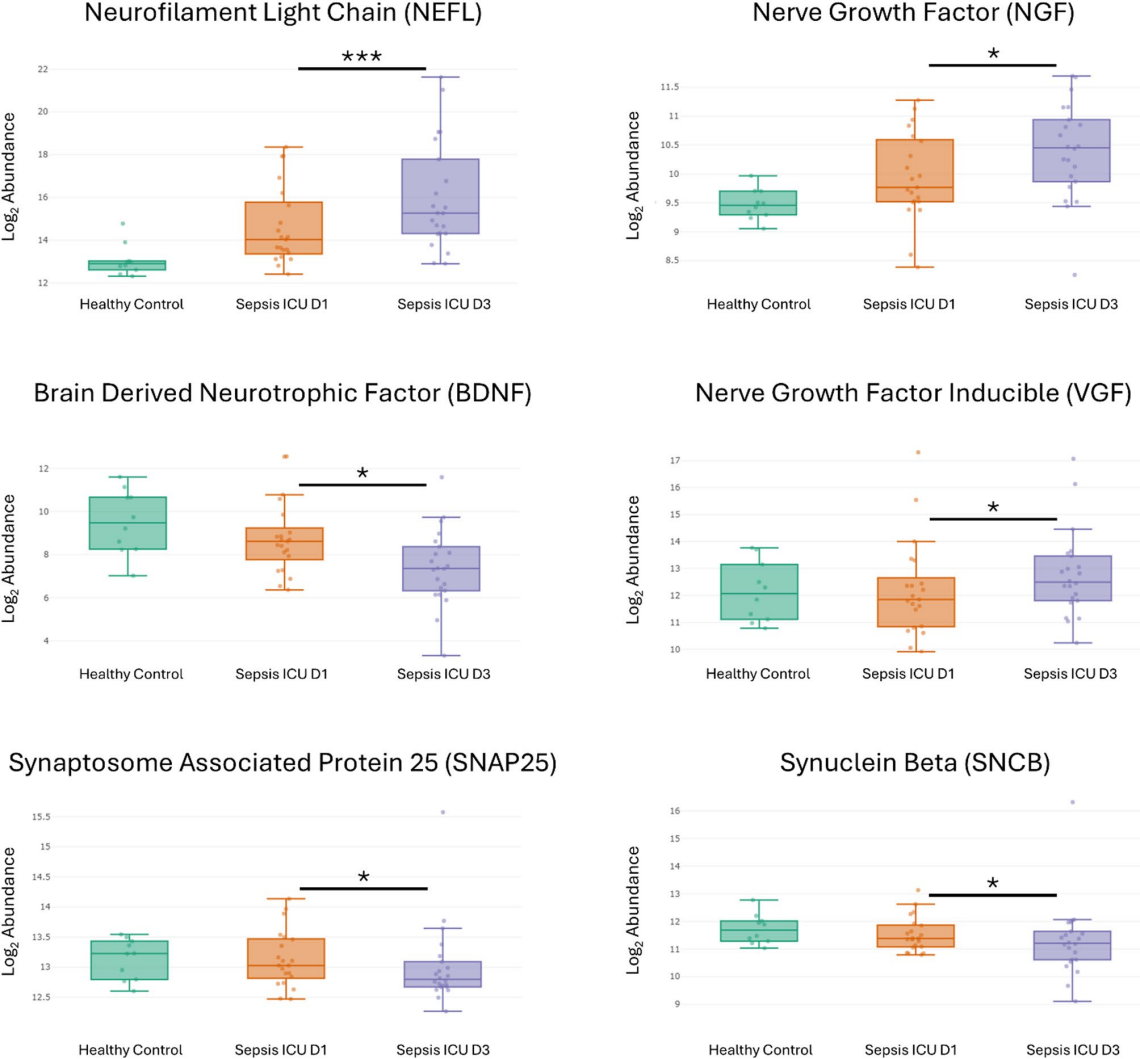
Box Plots illustrating the log2 abundance levels of six different brain-associated proteins across three groups: Healthy Control, Sepsis PICU D1, and Sepsis PICU D3. The proteins analyzed include Neurofilament Light Chain (NEFL), Nerve Growth Factor (NGF), Brain Derived Neurotrophic Factor (BDNF), Nerve Growth Factor Inducible (VGF), Synaptosome Associated Protein 25 (SNAP25), and Synuclein Beta (SNCB). The asterisk indicates the level of significance: 1 asterisk indicates a p<0.05, 2 asterisks a p<0.005, 3 asterisks a p<0.001. The horizontal line below the asterisks indicates the groups that have a significant difference in terms of median in the expression of proteins

Significant differences in brain-associated protein expression were observed between PICU D1 group and healthy controls (Fig. 4; adjusted p< 0.05) and between PICU D1 and D3 (Fig. 5; adjusted p< 0.05). CRH (FC −7.022; adj. p-value 1.15e-06) and NPY (FC −3.553; adj. p-value 1.15e-06) expressions were significantly reduced in the sepsis PICU D1 group, while NPY (FC 1.589; adj. p-value 0.0019) expression was elevated in the sepsis PICU D3 group compared to PICU D1. Neuronal Pentraxin 2 (NPTX2; FC 2.711; adj. p-value 0.0121), Neurofilament Heavy Chain (NEFH; FC 6.105; adj. p-value 0.0192), Microtubule Associated Protein Tau (MAPT; FC 2.728; adj. p-value 0.0197), and S100 Calcium Binding Protein B (S100B; FC 2.763; adj. p-value 0.0269) were significantly upregulated in the sepsis PICU D1 group compared to healthy controls, with NEFH expression further increased in PICU D3 (FC 1.808; adj. p-value 0.0112). In contrast, Neurogranin (NRGN; FC −2.122; adj. p-value 0.0364) and Neuronal Pentraxin Receptor (NPTXR; FC −1.814; adj. p-value 0.0398) expressions were higher in healthy controls than in the sepsis PICU D1 group. Additionally, NEFL (FC 2.165; adj. p-value 0.0006), Nerve Growth Factor (NGF; FC 1.134; adj. p-value 0.0260), and VGF Nerve Growth Factor Inducible (VGF; FC 1.499; adj. p-value 0.0318) expression were significantly increased in PICU D3 compared to PICU D1, whereas Brain-Derived Neurotrophic Factor (BDNF; FC −2.401; adj. p-value 0.0303), Synaptosomal-Associated Protein 25 (SNAP25; FC −1.193; adj. p-value 0.0333), and Synuclein Beta (SNCB; FC −1.395; adj. p-value 0.0377) expression were significantly lower in PICU D3.

### Clinical Outcome-Protein associations

**Fig. 6:**
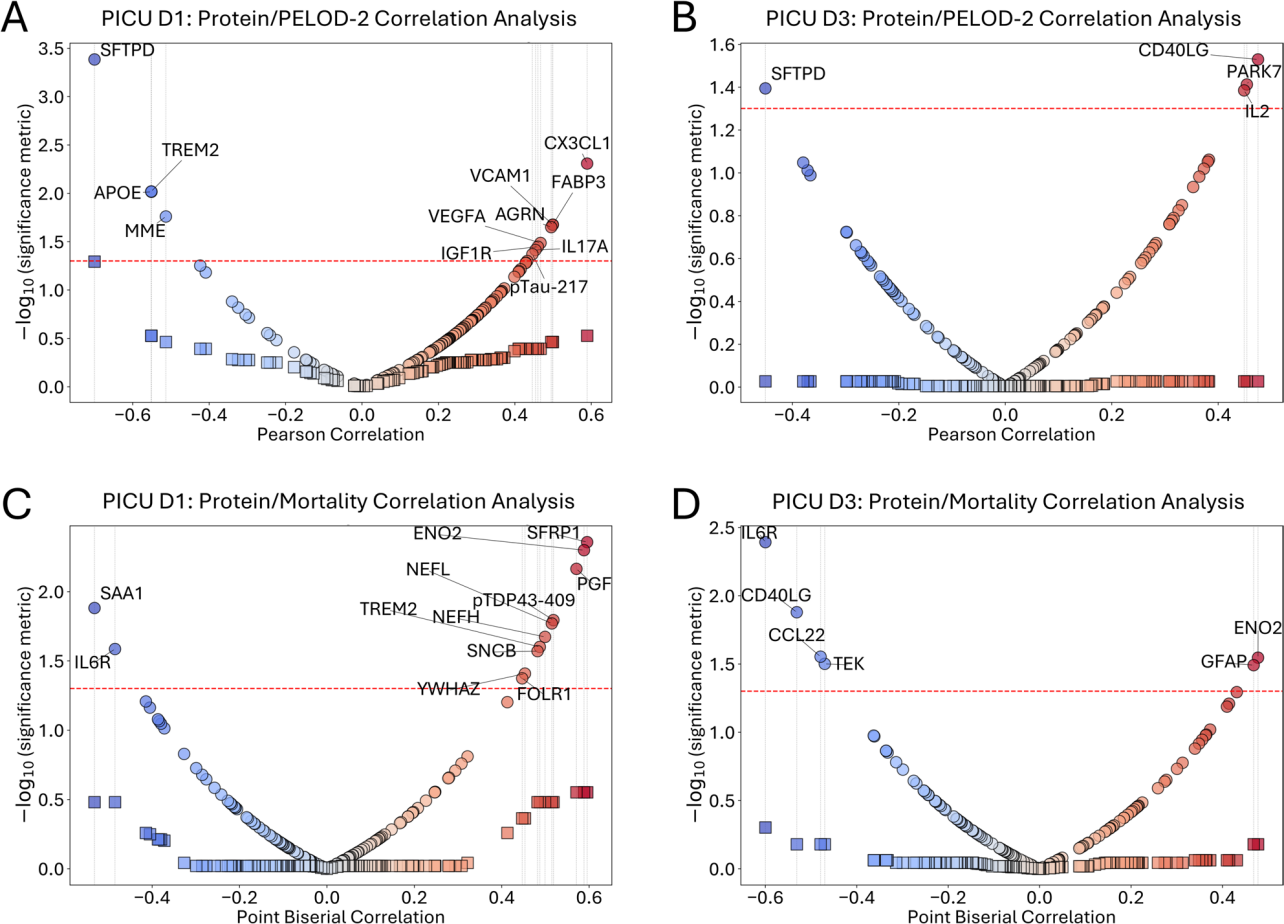
Associations between protein expression and clinical outcomes. **A** Pearson correlations between PELOD-2 scores and protein expression on PICU D1. **B** Pearson correlations between PELOD-2 scores and protein expression on PICU D3. **C** Point-biserial correlations between PICU D1 protein expression and mortality outcome **D** Point-biserial correlations between PICU D3 protein expression and mortality outcome. For all panels, the x-axis represents correlation coefficients, and the y-axis shows -log₁₀ transformed p-values. Points are color-coded by correlation direction (blue = negative correlation, red = positive correlation). Proteins with uncorrected p< 0.05 are labeled. Vertical reference lines connect proteins with significant uncorrected p-values to their corresponding false discovery rate (FDR)-adjusted significance levels. No correlations remained significant after FDR correction for multiple comparisons

Correlation analysis between protein expression and PELOD-2 scores revealed distinct patterns at each time point (Figs. 6A-B). On PICU D1, four proteins demonstrated negative correlations with PELOD-2 scores (SFTPD [FC −1.294; adj. p-value 0.5289], Triggering Receptor Expressed on Myeloid cells 2 [TREM2; FC 1.340; adj. p-value 0.2231], APOE [FC 1.069; adj. p-value 0.8217], and Membrane Metalloendopeptidase [MME; FC 8.739; adj. p-value 0.0030]), while eight proteins showed positive correlations (C-X3-C motif chemokine Ligand 1 [CX3CL1; FC 3.672; adj. p-value 7.44e-04], Fatty Acid Binding Protein 3 [FABP3; FC 5.788; adj. p-value 0.0072], Vascular Cell Adhesion Molecule 1 [VCAM1; FC 2.187; adj. p-value 0.0023], Agrin [AGRN; FC 1.201; adj. p-value 0.2792], Vascular endothelial Growth Factor A [VEGFA; FC 2.519; adj. p-value 5.92e-07], Insulin-like Growth Factor 1 Receptor [IGF1R; FC 1.167; adj. p-value 0.1917], IL17A [FC 8.897; adj. p-value 5.69e-04], and pTau-217 [FC 1.028; adj. p-value 0.9553]). By PICU D3, fewer significant associations were observed, with only SFTPD (FC 2.359; adj. p-value 3.94e-04) maintaining a negative correlation and three proteins (CD40 Ligand [CD40LG; FC −1.375; adj. p-value 0.1058], Parkinsonism Associated Deglycase [PARK7; FC −1.304; adj. p-value 0.0313], and IL-2; FC −3.241; adj. p-value 0.0066) exhibiting positive correlations with PELOD-2 scores.

Point-biserial correlation analysis identified multiple proteins associated with mortality outcomes (Figs. 6C-D). On PICU D1, two proteins (SAA1 [FC 563.228; adj. p-value 1.36e-27] and IL6R [FC −2.273; adj. p-value 0.0384]) were negatively correlated with mortality, while ten proteins (SFRP1 [Secreted Frizzled-Related Protein 1; FC 1.996; adj. p-value 0.2905], ENO2 [neuron-specific enolase 2; FC 1.080; adj. p-value 0.8217], PGF [Placental Growth Factor; FC 1.346; adj. p-value 0.2259], pTDP43-409 [phosphor-Translocated in sarcoma/DNA-binding protein 43–409; FC −1.056; adj. p-value 0.8313], NEFL [FC 2.946; adj. p-value 0.0832], NEFH [FC 6.105; adj. p-value 0.0192], TREM2 [FC 1.340; adj. p-value 0.2231], SNCB [FC −1.140; adj. p-value 0.7293], YWHAZ [Tyrosine 3-Monooxygenase/Tryptophan 5-Monooxygenase Activation Protein, Zeta Polypeptide; FC 1.013; adj. p-value 0.9942], and FOLR1 [Folate Receptor 1; FC 1.329; adj. p-value 0.1013] showed positive correlations. PICU D3 analysis revealed four proteins with negative mortality correlations (IL6R [FC 2.079; adj. p-value 5.59e-04], CD40LG [FC −1.193; adj. p-value 0.1058], C-C motif chemokine ligand 22 [CCL22; FC −1.387; adj. p-value 0.1100], and receptor tyrosine kinase [TEK; FC 1.094; adj. p-value 0.1981]) and two with positive correlations (ENO2 [FC −1.418; adj. p-value 0.0447] and Glial Fibrillary Acidic Protein [GFAP; FC 1.110; adj. p-value 0.7128]).

### Enriched signaling pathways

**Fig. 7:**
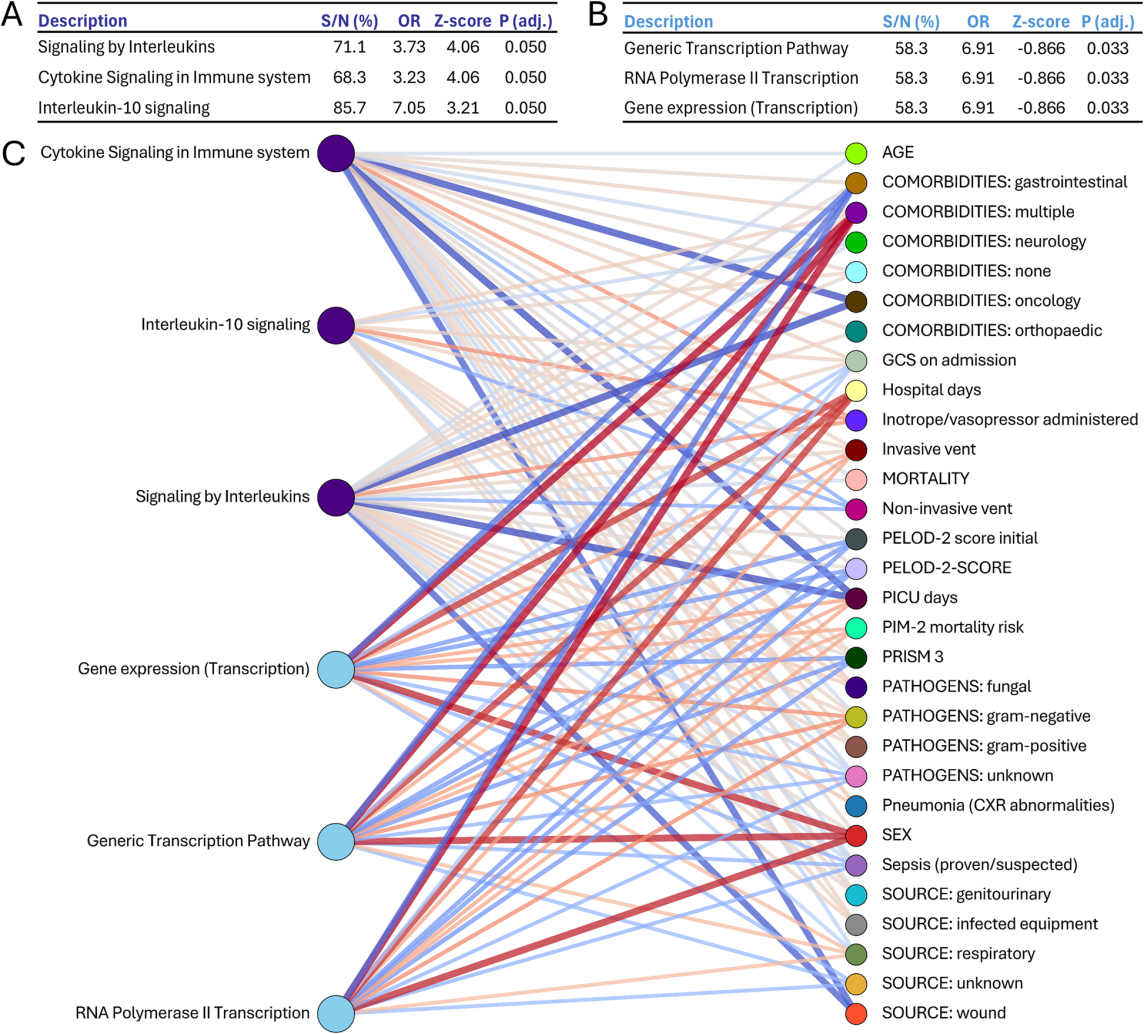
Functional Analysis showing enriched pathways in Over-Representation Analysis (ORA) and a network diagram displaying the associations between the top enriched pathways identified through ORA analysis and clinical features. **A** A list that shows the pathways that are significantly enriched when comparing sepsis PICU D1 to the healthy controls. The pathways are ranked by their significance, with associated values, highlighting the differences in pathway activity linked to early sepsis. **B** A list that shows the pathways that are significantly enriched when comparing sepsis PICU D1 to sepsis PICU D3 patients. The pathways are ranked by their significance, with associated values, highlighting the differences in pathway activity linked to continuing sepsis. **C** The associations between significant Reactome pathways and clinical variables. The colour indicates the relative strength of the association, with blue indicating a negative correlation and red indicating a positive correlation. The line thickness is proportional to the strength of the correlation

In PICU D1 patients, “Signaling by Interleukins”, “Cytokine Signaling in Immune System”, and “Interleukin-10 signaling” were significantly enriched and upregulated compared to healthy controls (OR: 3.73, Z-score: 4.06; OR: 3.23, Z-score: 4.06; OR: 7.05, Z-score: 3.21, respectively; all adjusted p ≤ 0.05) (Fig. 7A). In contrast, pathways related to “Generic Transcription Pathway”, “RNA Polymerase II Transcription”, and “Gene expression (Transcription)” were downregulated by PICU D3 (OR: 6.91, Z-score: −0.866 for all three pathways; adjusted p≤ 0.05) (Fig. 7B). Associations between clinical variables and pathway proteins are shown in Supplementary Figs. 4–9, with box colors representing correlation coefficients (p< 0.05).

Correlation analysis showed that “Cytokine Signaling in Immune system” and “Signaling by Interleukins” pathways were significantly reduced in oncological patients, with decreased PICU days and wound infections (Fig. 7C). In contrast, “Gene expression (Transcription)”, “Generic Transcription Pathway”, and “RNA Polymerase II Transcription” were elevated in patients with multiple comorbidities, longer hospital stays, and in males, but decreased in patients with gastrointestinal comorbidities (Fig. 7C).

### Protein-Protein interactions

**Fig. 8:**
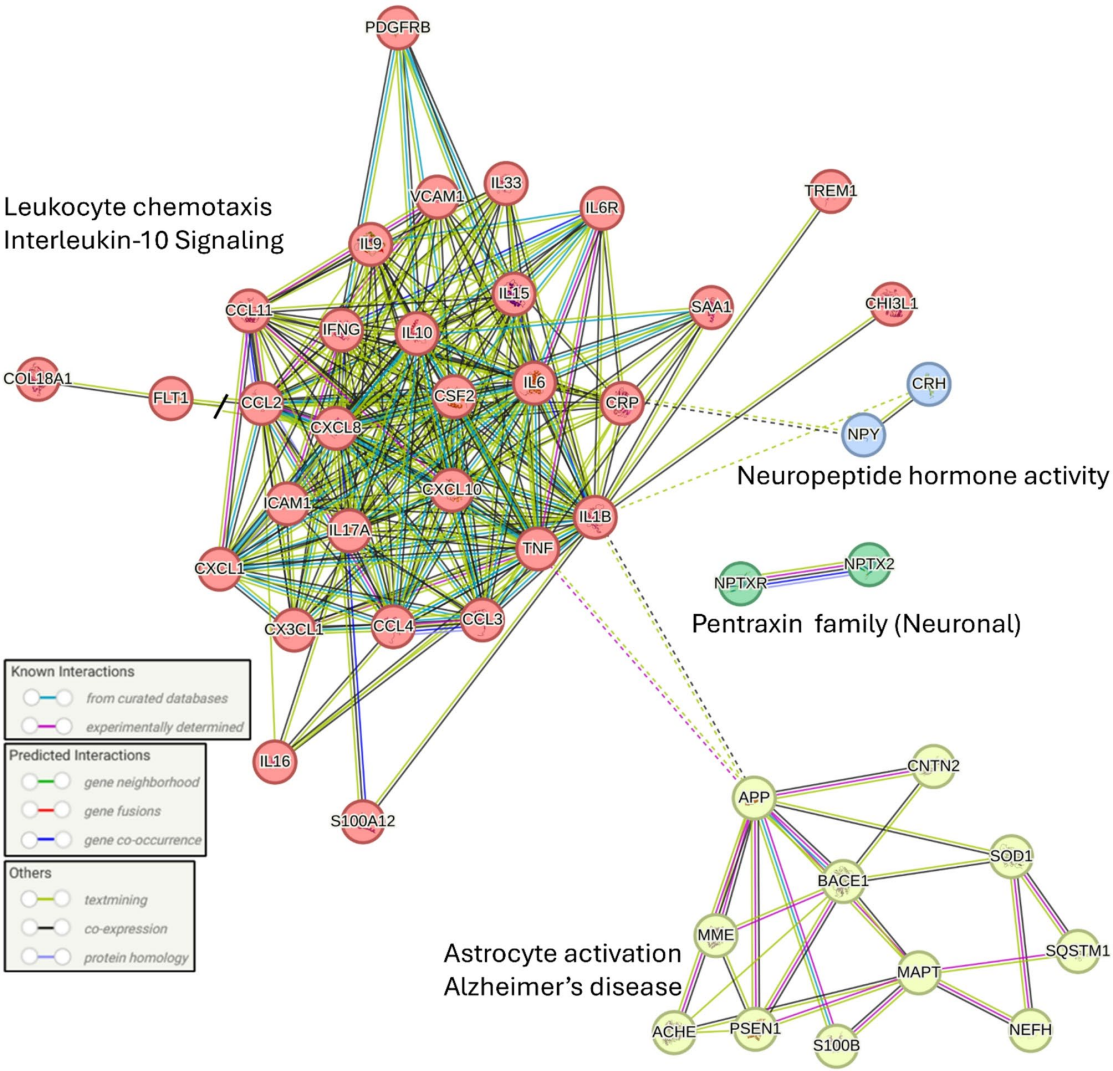
Protein-protein interaction network of differentially expressed proteins in pediatric sepsis reveals distinct functional clusters. STRING analysis of significantly dysregulated proteins identified through proteomic profiling shows a highly interconnected network with functional clustering. Nodes represent individual proteins, with node colors indicating different functional groups. Edges represent protein-protein interactions with different line types and colors indicating the source of interaction evidence as shown in the legend. The network reveals several distinct functional clusters including a large inflammatory response cluster centered around cytokines and chemokines (IL-9, IL-10, IL-6, TNF, CXCL8, CCL14, etc.) that is associated with "Leukocyte chemotaxis" and "Interleukin-10 Signaling" pathways. A smaller brain-associated cluster includes proteins involved in "Neuropeptide hormone activity" (CRH, NPY), "Pentraxin family (Neuronal)" proteins (NPTX2, NPTXR), and "Astrocyte activation/Alzheimer's disease" pathways (APP, BACE1, MAPT, PSEN1, SOD1, S100B, MME, ACHE [Acetylcholinesterase], NEFH, SQSTM1 [Sequestosome 1], CNTN2 [Contactin 2]). TNF and IL-1β appear to bridge inflammatory and neurological clusters, suggesting potential crosstalk between peripheral inflammation and brain-associated pathways in pediatric sepsis

Protein-protein interaction network analysis using STRING revealed a highly interconnected network of 45 differentially expressed proteins with significant functional clustering (Fig. 8; PPI enrichment p< 0.001). The network demonstrated two primary functional modules: a large inflammatory cluster dominated by cytokines and chemokines (e.g., IL6, IL9, IL10, TNF, CXCL8 [C-X-C motif chemokine ligand 8], CCL14) associated with leukocyte chemotaxis and interleukin-10 signaling pathways, and a distinct brain-associated cluster encompassing proteins involved in neuropeptide hormone activity (CRH, NPY), pentraxin family neuronal proteins (NPTX2, NPTXR), and astrocyte activation/Alzheimer’s disease pathways (e.g., APP [Amyloid Precursor Protein], BACE1 [Beta-site Amyloid precursor protein Cleaving Enzyme 1], MAPT, PSEN1 [Presenilin 1], SOD1 [Superoxide dismutase]). Notably, TNF and IL-1 β served as bridging proteins between these clusters, suggesting potential crosstalk between systemic inflammation and neurological processes in pediatric sepsis. This network topology supports the identification of novel brain-associated biomarkers that may reflect neuroinflammatory processes during septic episodes in children.

### Disease enrichment

**Fig. 9:**
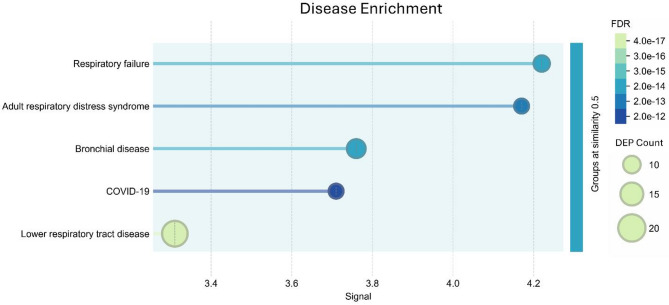
Disease enrichment analysis revealed significant respiratory pathology in the pediatric sepsis proteome. The x-axis represents the signal strength (enrichment score), while bubble size indicates DEP count (number of proteins associated with each disease category). Color intensity represents the false discovery rate (FDR), with lighter colors indicating higher significance (lower FDR values). The most significantly enriched disease categories include respiratory failure and adult respiratory distress syndrome (ARDS), followed by bronchial disease, COVID-19, and lower respiratory tract disease. FDR values range from 4.50e-17 to 2.09e-12, with gene counts ranging from approximately 8 to 18 proteins per disease category

DISEASES database enrichment analysis revealed significant over-representation of respiratory pathology-associated genes among the differentially expressed proteins in pediatric sepsis (Fig. 9). The most significantly enriched disease categories were respiratory failure (FDR = 4.58e-14, 9 DEPs) and adult respiratory distress syndrome (FDR = 1.52e-13, 8 DEPs), both showing high signal strength (> 4.0). Additional respiratory-related enrichments included bronchial disease (FDR = 4.75e-14, 11 DEPs), COVID-19 (FDR = 2.09e-12, 8 DEPs), and lower respiratory tract disease (FDR = 4.50e-17, 18 DEPs). These findings demonstrate that the proteomic signature of pediatric sepsis is strongly associated with respiratory dysfunction and acute lung injury pathways, consistent with the clinical manifestations of sepsis-associated organ failure in children.

## Discussion

This study characterized plasma protein expression profiles in pediatric patients with sepsis, comparing them to age- and sex-matched healthy controls. Additionally, a longitudinal analysis of protein expression patterns was conducted for sepsis patients during their hospitalization in the PICU. Signaling pathways were identified and correlated with clinical parameters. Moreover, novel brain-associated protein expression patterns were identified.

The demographic characteristics and illness severity of our study population were consistent with those of previously published pediatric sepsis studies (Mickiewicz et al. 2018; Zurek and Vavrina 2015), supporting the generalizability of our findings to the broader pediatric sepsis population. Our confirmation that bacterial pathogens remained the predominant cause of sepsis, with gram-positive bacteria being most common and pulmonary infections serving as the primary source (Weiss and Fitzgerald 2024; Weiss et al. 2015; Simmons et al. 2012; Randolph and McCulloh 2014), validated our study design and patient selection criteria. The alignment of our treatment protocols with current clinical guidelines (Simmons et al. 2012; Weiss et al. 2020) ensured that our outcomes reflected contemporary standard-of-care practices, making our results applicable to current clinical settings.

### Differentially expressed proteins

Among the 40 leading differentially expressed proteins identified at PICU D1, RFC combined with Boruta feature selection identified 29 proteins that demonstrated robust discriminatory capacity between PICU D1 sepsis patients and healthy controls, suggesting their potential utility as diagnostic biomarkers. Cytokine-related pathways were highly represented in sepsis patients, which exhibited significant enrichment on PICU D1, and constituted a fundamental component of SIRS (Niu et al. 2023). The concurrent upregulation of pro-inflammatory and anti-inflammatory pathways during the initial phase reflects the orchestrated immune response, with anti-inflammatory mechanisms serving to modulate excessive inflammatory activation (Monneret et al. 2008). This complex interplay involved key pro-inflammatory mediators, including IL-6, IL-1, and TNF (Krüttgen and Rose-John 2012), while elevated IL-17 production promoted the expression of additional pro-inflammatory factors such as Chemokines: CXCL1, CXCL2, CXCL8, IL-2, and IL-8 (Ge et al. 2020). Interleukin 10, traditionally recognized for its immunosuppressive properties, provided pleiotropic effects, including enhancement of CD8 + T cell function and pro-inflammatory activity during inflammatory states (Saxton et al. 2021). The observed correlations between differentially expressed proteins and clinical parameters suggested potential prognostic value for disease severity, pathogen classification, infection source identification, and therapeutic response prediction.

### Clinical Outcome-Protein associations

The observed correlations between protein expression and clinical outcomes revealed potentially meaningful biological patterns. The negative correlation of SFTPD with both PELOD-2 scores and mortality aligned with its established role as a biomarker of lung injury severity in pediatric critical illness, where elevated plasma SP-D levels were associated with severe pediatric Acute Respiratory Distress Syndrome (ARDS) and poor outcomes in children with acute respiratory failure (Dahmer et al. 2020). The PELOD-2 score itself has demonstrated robust predictive validity for mortality in critically ill children, with studies showing area under the ROC curve values ranging from 0.75 to 0.89 across diverse pediatric intensive care populations (Leteurtre et al. 2015). The positive correlation observed between ENO2 and mortality on both D1 and D3 supported its recognized utility as a biomarker of neuronal injury, as elevated ENO2 levels in serum and cerebrospinal fluid have been consistently associated with poor outcomes in pediatric brain injury, with meta-analyses demonstrating significantly higher ENO2 levels in children with unfavorable outcomes following traumatic brain injury (Nakhjavan-Shahraki et al. 2017). Similarly, GFAP’s positive correlation with mortality on D3 was consistent with its established role as a brain injury biomarker, where elevated plasma GFAP levels have been shown to predict both acute neurologic injury and death in pediatric patients (Fraser et al. 2011; Bembea et al. 2011).

### Temporal dynamics of differentially expressed proteins and signaling pathways

Temporal analysis of protein expression patterns revealed 34 differentially expressed proteins between PICU D1 and D3. RFC with Boruta feature selection identified 9 proteins with discriminatory potential between these time points, albeit with limited clustering efficiency. This reduced discriminatory capacity suggested that proteomic biomarkers may have diminished utility in distinguishing different temporal phases of sepsis. The early inflammatory response at PICU D1 was followed by significant depression of generic transcription, RNA Polymerase II transcription, and gene expression pathways, when compared to PICU D3. This temporal pattern suggested that peak transcriptomic responses typically manifested early, and they are associated with systemic inflammation, followed by progression to an immunosuppressive state (Calvano et al. 2005; Talwar et al. 2006; Chang et al. 2014). The subsequent transcriptional suppression likely reflected multiple physiological adaptations, including cellular exhaustion following intense inflammatory activation, development of protective cellular hibernation mechanisms, enhanced protein catabolism for energy generation, and potential endotoxin tolerance manifesting as diminished cellular responsiveness to sustained inflammatory stimuli (Adib-Conquy et al. 2003; Christaki et al. 2011; Biolo et al. 1997).

Analysis of correlations between sepsis-associated signaling pathways and clinical variables revealed attenuated cytokine/interleukin signaling in patients with oncological comorbidities. This immunological modulation may be attributed to cancer-induced immune alterations and therapeutic interventions (Williams et al. 2023). While neutropenic sepsis remains a leading cause of mortality in patients receiving antineoplastic therapy (Vioral and Wentley 2015), there is limited evidence for substantial differences in proinflammatory factor production between oncological and non-oncological populations (Kochanek et al. 2019).

### Novel Brain-Associated biomarkers

The neuroimmune axis mediates systemic inflammation through complex mechanisms critical for therapeutic development (Wheway et al. 2007). Analysis revealed decreased CRH at PICU D1, contradicting typical stress-induced HPA axis activation patterns, though corticosteroid efficacy in sepsis remains controversial (Ilias et al. 2023; Ngo Ndjom et al. 2017). NPY, a vasoconstrictor and orexigenic hormone (Hauser et al. 1995), showed initial reduction at D1 followed by elevation at D3. The concurrent early reduction in both CRH and NPY suggested impaired central stress response mechanisms. Reduced CRH compromises HPA axis function while decreased NPY impairs stress resilience, potentially contributing to acute and chronic neurological dysfunction in sepsis (Wheway et al. 2007; Taylor et al. 2010; Claes 2004).

Sepsis induced concurrent dysregulation of key brain cellular proteins. At PICU D1, significant upregulation occured in NEFH (neuronal cytoskeletal component) (Marriott et al. 2024), MAPT (neuronal microtubule stabilizer) (Buchholz and Zempel 2024), and S100b (astrocyte calcium-binding inflammatory mediator) (Hu et al. 2023). NEFH and NEFL expression progressively increased through PICU D3, indicating ongoing axonal injury (Zhong et al. 2020; Abdullah et al. 2025; Gaetani et al. 2019). Elevated NEFH alters axonal architecture and transport (Hoffman et al. 1987), while increased MAPT disrupts microtubule dynamics and promotes tau hyperphosphorylation (Strang et al. 2019). Astrocytic S100b elevation triggers neuroinflammatory cascades and blood-brain barrier dysfunction (Sorci et al. 2010; Lipcsey et al. 2010). NEFL upregulation compounds these effects by further compromising axonal integrity (Gaetani et al. 2019), creating a complex pathophysiological cascade of neuronal dysfunction.

Synaptic proteins exhibited temporal expression changes, with SNAP25 and SNCB showing peak expression at PICU D1. During sepsis, SNAP25 dysregulation impairs neurotransmitter release by disrupting synaptic vesicle fusion, SNARE complex assembly, and calcium channel function (Noor and Zahid 2017). Concurrent decrease in SNCB compromises synaptic vesicle dynamics, alpha-synuclein homeostasis, and dopamine regulation while reducing protection against protein aggregation (George 2002; Ninkina et al. 2021). Synaptic dysfunction manifests through multiple mechanisms: impaired presynaptic vesicle release, disrupted postsynaptic AMPA receptor trafficking via NPTX2/NPTXR dysfunction (Xiao et al. 2017), and altered NRGN-dependent calcium signaling. This comprehensive synaptic failure during sepsis may contribute to SAE (Dumbuya et al. 2023).

Analysis of neurotrophic factors during sepsis revealed BDNF deficiency, impairing neuronal survival, synaptic plasticity, and neurogenesis (Tsutsui et al. 2023; Shi and Hu 2011; Cordeiro et al. 2016; Colucci-D’Amato et al. 2020). Concurrent NGF elevation enhances neuronal survival via TrkA-mediated pathways and increases nociceptive sensitivity (Ebendal 1989; McMahon 1996; Marlin and Li 2015). VGF upregulation provides partial compensation through enhanced synaptic plasticity and neurogenesis (Soliman et al. 2019; Ferri et al. 2011). Despite NGF and VGF compensatory mechanisms, persistent functional impairment likely occurs due to BDNF’s critical role in adult neuroplasticity. These neurotrophic alterations may contribute to lingering cognitive deficits observed in some pediatric sepsis survivors (Ravikumar et al. 2022).

### Protein-Protein interactions

The protein-protein interaction network revealed TNF and IL-1β as critical bridging proteins linking inflammatory and neurological processes in pediatric sepsis. This topology aligned with established pathophysiology where TNF-α and IL-1β served as central mediators, with TNF-α concentrations approximately 10-fold higher in sepsis patients and correlating with mortality (Shakoory et al. 2016). The distinct brain-associated protein cluster, including neuropeptide hormones, astrocyte activation markers, and Alzheimer’s disease-related proteins, suggested neuroinflammatory processes like neurodegenerative conditions where microglial and astrocyte activation leads to excessive cytokine secretion (Glass et al. 2010). This supported emerging evidence that sepsis-associated brain inflammation may progress to chronic neuroinflammation, with IL-1β playing a critical role in the acute-to-chronic transition through NLRP3 (NOD-Like Receptor family Pyrin domain containing 3) inflammasome activation (Zhao et al. 2020).

### Disease enrichment

The disease enrichment analysis demonstrated a respiratory-centric proteomic signature, with respiratory failure and ARDS as the most significantly over-represented pathological processes. This finding reflected the clinical reality that ARDS occurs in 15–30% of severely ill children and represents a leading cause of mortality in pediatric sepsis (Khemani et al. 2015). The clinical relevance of these findings was underscored by the observation that chest X-ray abnormalities were present in 91% of the patient population under study, highlighting the predominant respiratory involvement in pediatric sepsis. The strong COVID-19 association reflected shared pathophysiological mechanisms between viral and bacterial sepsis-induced respiratory failure, involving similar inflammatory cascades and alveolar-capillary barrier disruption (Matthay et al. 2019). Proteomic studies have shown that DNA-bound proteins and nucleosomes are over-represented in pediatric ARDS, serving as damage-associated molecular patterns independently associated with mortality (Yehya et al. 2022). These findings aligned with pediatric anatomical vulnerabilities, including immature alveolar development and increased susceptibility to inflammatory-mediated respiratory dysfunction (Rimensberger 2015).

### Limitations

Our comprehensive proteomic analysis has elucidated multiple biomarkers characterizing the inflammatory response in sepsis, providing crucial insights into key molecular pathways, their temporal evolution, and potential therapeutic targets. Notably, this investigation represented the first study of novel brain-associated protein expression patterns, exploring their potential utility as diagnostic biomarkers and their implications for sepsis management. However, several methodological limitations warrant consideration. First, the study’s generalizability is constrained by its single-center design and limited sample size. Second, the restricted observation period of three days may not fully capture the complete temporal dynamics of protein expression in sepsis. Third, the sepsis criteria used in this study were derived from the International Consensus Criteria for Pediatric Sepsis and Septic Shock (Schlapbach et al. 2024; Goldstein et al. 2005). While these sepsis criteria are still relevant, future work should also consider the Phoenix sepsis criteria (Sanchez-Pinto et al. 2024). Fourth, the clinical outcome-protein associations were statistically significant by raw p-values, but not after FDR correction, likely reflecting the study’s sample size limitations and the inherent challenge of detecting biomarker associations in heterogeneous critically ill pediatric populations. Fifth, while our temporal analysis demonstrated consistent population-level changes from inflammatory activation to transcriptional suppression, future investigations that focus on patient-specific trajectory analysis could offer enhanced clinical utility. Identifying molecular patterns linked to clinical improvement or deterioration may help reveal subgroups with distinct therapeutic needs. Sixth, the absence of an external validation cohort limits our ability to confirm the generalizability of the identified biomarker associations across different pediatric sepsis populations and clinical settings. Despite these caveats, the data are worthy of reporting and further exploration. Future investigations should prioritize multicenter study designs with expanded cohort sizes, extended observation periods, and independent validation cohorts to enhance the comprehensive understanding of sepsis pathophysiology and optimize therapeutic strategies.

## Conclusions

This study identified distinct protein expression signatures that differentiate sepsis patients from healthy controls and characterized temporal patterns during PICU hospitalization. Protein-protein interaction network analysis revealed TNF and IL-1β as critical bridging proteins linking systemic inflammation to neurological processes, while disease enrichment analysis demonstrated a respiratory-centric proteomic signature with significant over-representation of respiratory failure and acute respiratory distress syndrome pathways. Although multiple proteins correlated with PELOD-2 scores and mortality, statistical significance was lost after multiple comparison correction, highlighting the challenge of identifying robust clinical biomarkers in heterogeneous pediatric populations. Brain-associated protein expression showed differential patterns, with temporal variations linked to PICU length of stay, potentially contributing to SAE (Dumbuya et al. 2023) and cognitive impairment in sepsis survivors (Ravikumar et al. 2022). While these molecular signatures hold promise as diagnostic and prognostic biomarkers, the complex pathophysiology of sepsis challenges single-biomarker approaches (Leonard et al. 2024). The identification of mechanistic connections between inflammation, neurological dysfunction, and respiratory pathology suggests that multiplexed biomarker panels targeting these interconnected pathways may improve diagnostic accuracy, though practical limitations could hinder widespread clinical use (Liu et al. 2014).

## Supplementary Information

Supplementary Material 1.

## Data Availability

The datasets generated and/or analysed during the current study are available from the corresponding author on reasonable request.
