# Supplementary material for: Differential protein expression and enriched pathways in pediatric sepsis: identification of novel brain-associated biomarkers revealed through proteomic profiling
Source: Mol Med. 2025 Nov 26;32:14. doi: 10.1186/s10020-025-01397-x (PMC12875032; doi:10.1186/s10020-025-01397-x)

SUPPLEMENTARY FIGURE LEGENDS

Supplementary Figure 1. Feature comparisons, feature importances and dimensionality reduction. A) Shared features across classifiers when predicting Health Control vs Sepsis PICU D1. The decision tree methods, when assembled into ensembles, were useful for determining the important features that predicted class membership when working with this high dimensional data. These methods include Random Forest Classifier (RFC), Extremely Randomized Trees Classifier (ETC) and Gradient Boosting Classifier (GBC, although this is not a decision tree method). Combined with Boruta feature selection methods, these different classifiers were used to identify the most important features that predict class membership. Note that the GBC model identified 7 features as important that were common to both the RFC and ETC models. RFC was ultimately chosen as it is a more conservative model that incorporated the most features. **B)** A relatively small set of protein features separated health controls from patients with sepsis PICU D1. Left: Feature importances calculated using RFC and Boruta feature selection process. The top 20 features are shown out of a total of 29 important features. Right: the top 29 features readily separate the classes. **C)** A relatively small set of protein features separated sepsis PICU D1 versus D3. Left: Feature importances calculated using RFC and Boruta feature selection process. Nine features were selected as important. Right: these nine features somewhat separate the classes.

Supplementary Figure 2. Associations between Clinical Variables and DEPs: Sepsis PICU D1 versus HC. All the differentially expressed proteins that provided the basis for the curated Reactome pathways were compared with the clinical variables. The associations were determined with either a Pearson's correlation coefficient for continuous/continuous comparisons or a point biserial correlation coefficient for binary/continuous comparisons. Note that some

features (e.g., source of pathogen, pathogen type, and comorbidities) had multiple values and were converted to one-hot encoded binary features. Boxes are colored by correlation coefficient value and are marked ** for a p-value < 0.05.

Supplementary Figure 3. Associations between Clinical Variables and DEPs: Sepsis PICU D1 versus D3. All the differentially expressed proteins that provided the basis for the curated Reactome pathways were compared with the clinical variables. The associations were determined with either a Pearson's correlation coefficient for continuous/continuous comparisons or a point biserial correlation coefficient for binary/continuous comparisons. Note that some features (e.g., source of pathogen, pathogen type, and comorbidities) had multiple values and were converted to one-hot encoded binary features. Boxes are colored by correlation coefficient value and are marked ** for a p-value < 0.05.

Supplementary Figures 4-6. Associations between Clinical Variables and Reactome Pathways: Sepsis PICU D1 versus HC. The differentially expressed proteins that provided the basis for the 3 inflammatory pathways curated Reactome were compared with the clinical variables. The associations were determined with either a Pearson's correlation coefficient for continuous/continuous comparisons or a point biserial correlation coefficient for binary/continuous comparisons. Note that some features (e.g., source of pathogen, pathogen type, and comorbidities) had multiple values and were converted to one-hot encoded binary features. Boxes are colored by correlation coefficient value and are marked ** for a p-value < 0.05.

Supplementary Figures 7-9. Associations between Clinical Variables and Reactome Pathways: Sepsis PICU D1 versus D3. The differentially expressed proteins that provided the

basis for the 3 transcription pathways curated Reactome were compared with the clinical variables. The associations were determined with either a Pearson's correlation coefficient for continuous/continuous comparisons or a point biserial correlation coefficient for binary/continuous comparisons. Note that some features (e.g., source of pathogen, pathogen type, and comorbidities) had multiple values and were converted to one-hot encoded binary features. Boxes are colored by correlation coefficient value and are marked ** for a p-value < 0.05.

Supplementary Figure 1.

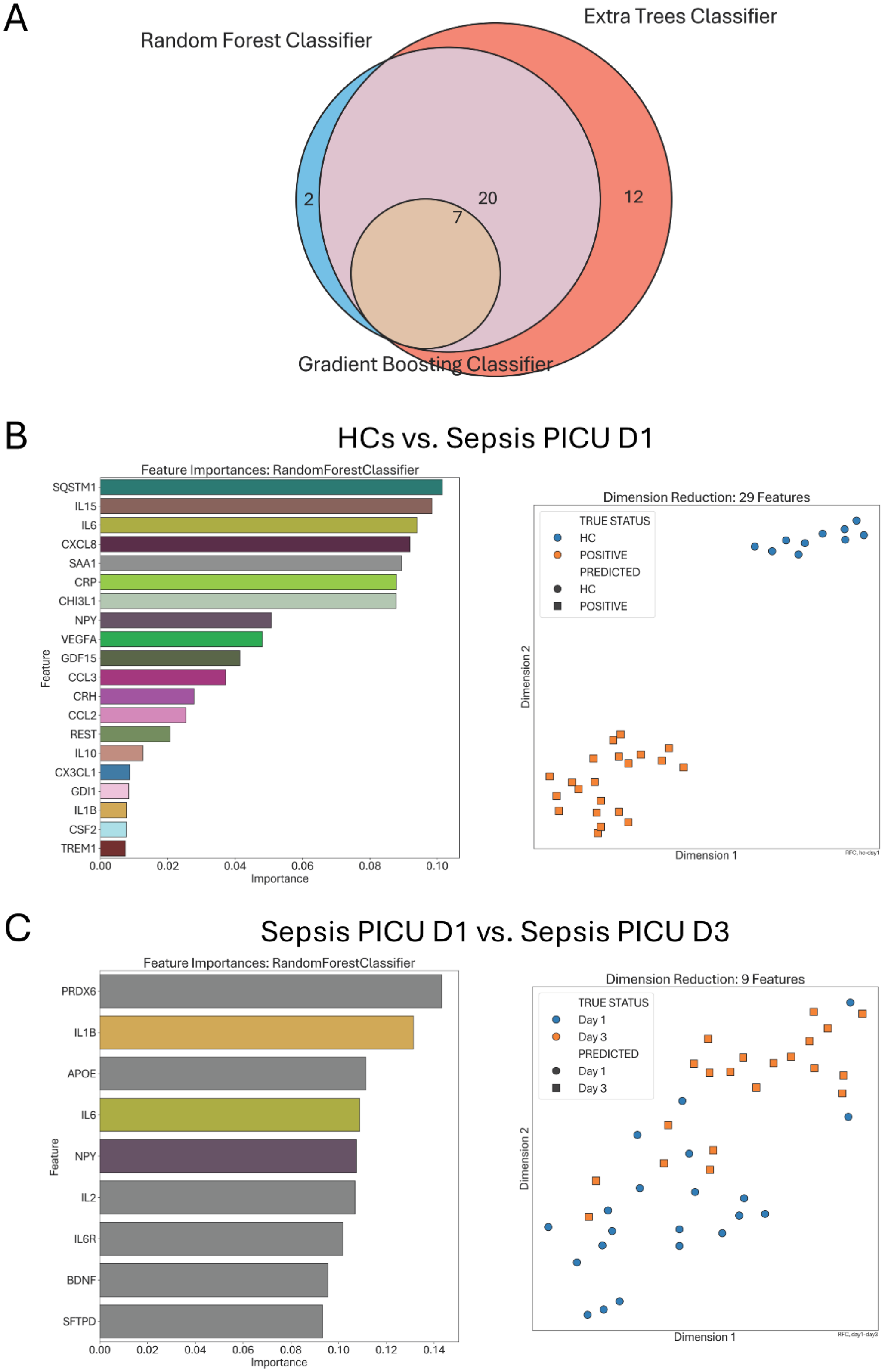

Supplementary Figure 2.

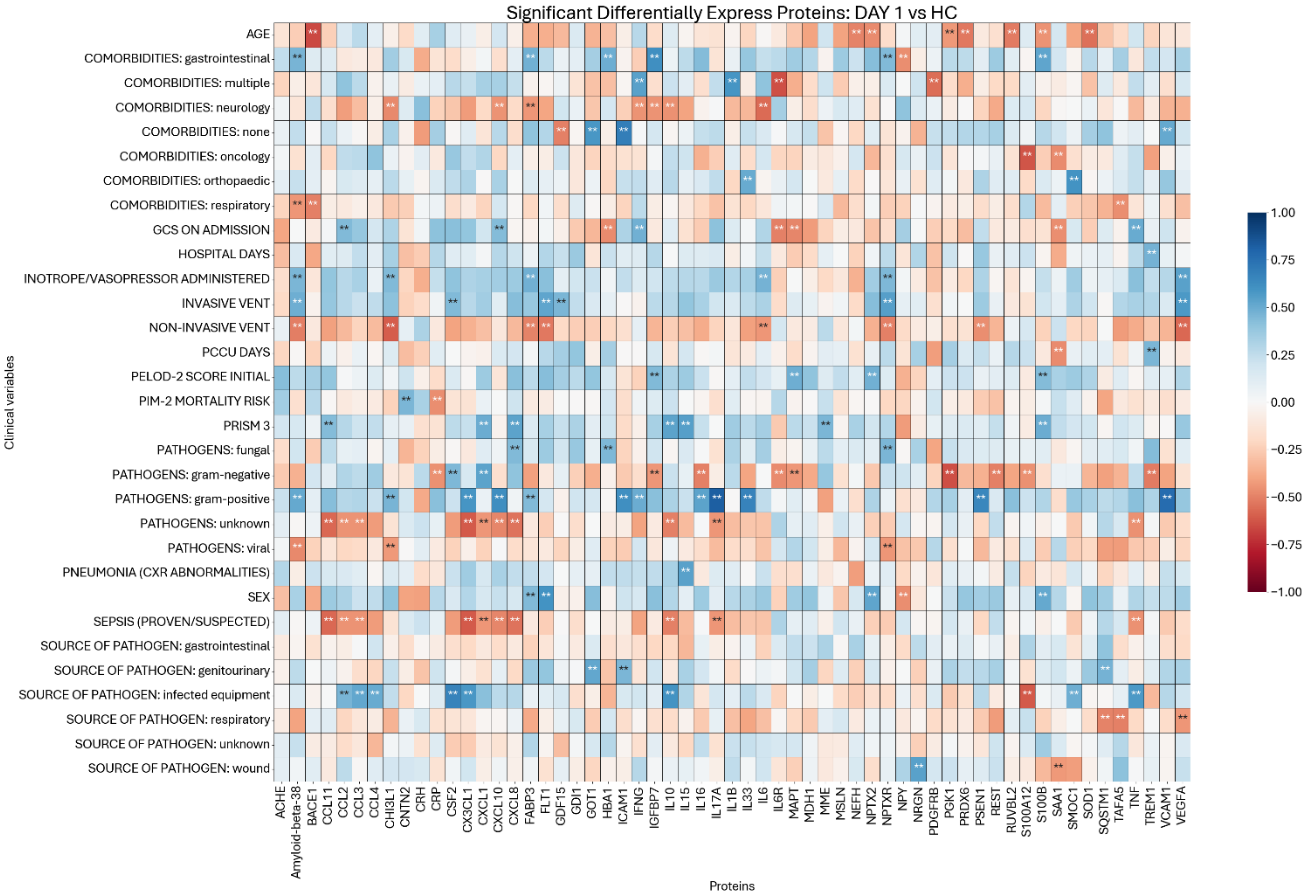

Supplementary Figure 3.

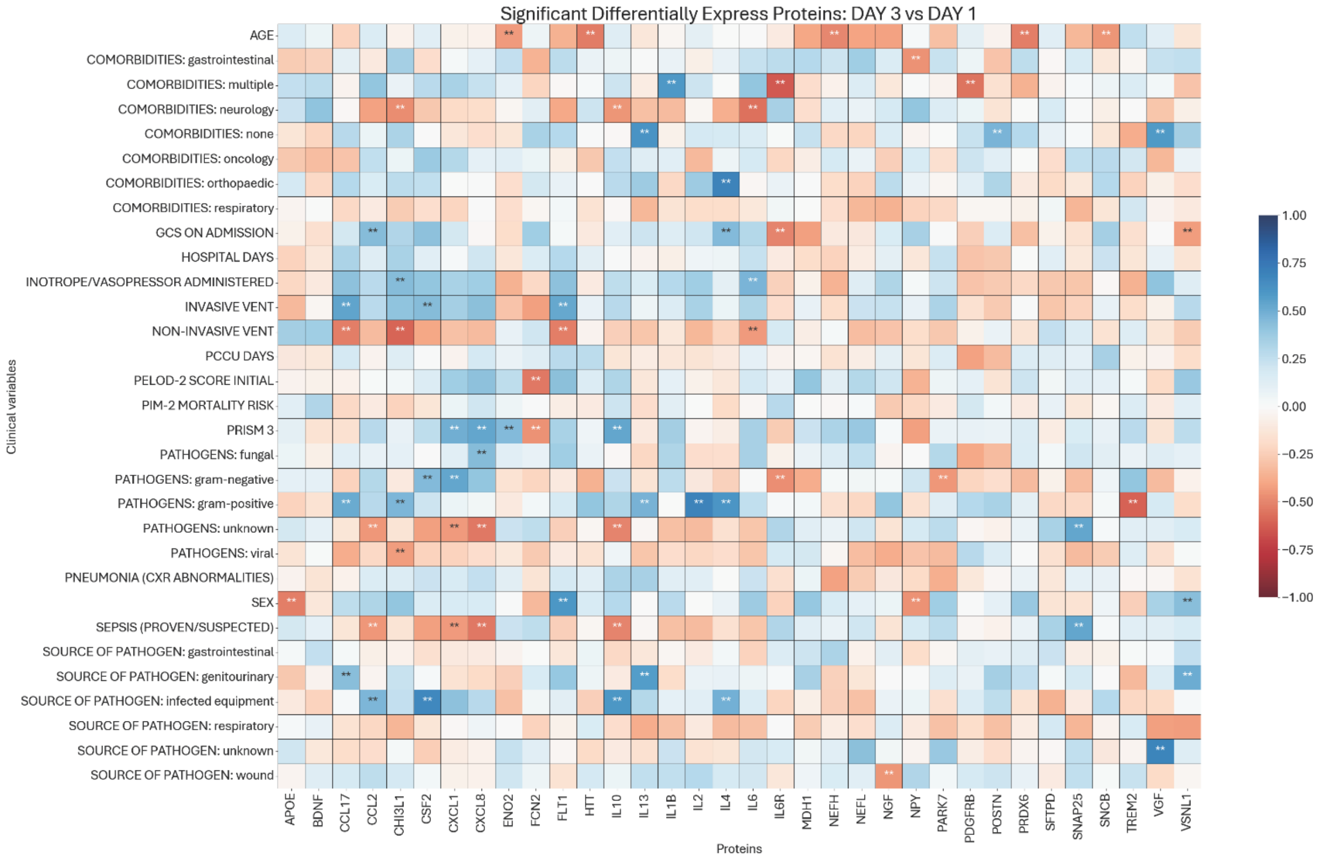

Supplementary Figure 4.

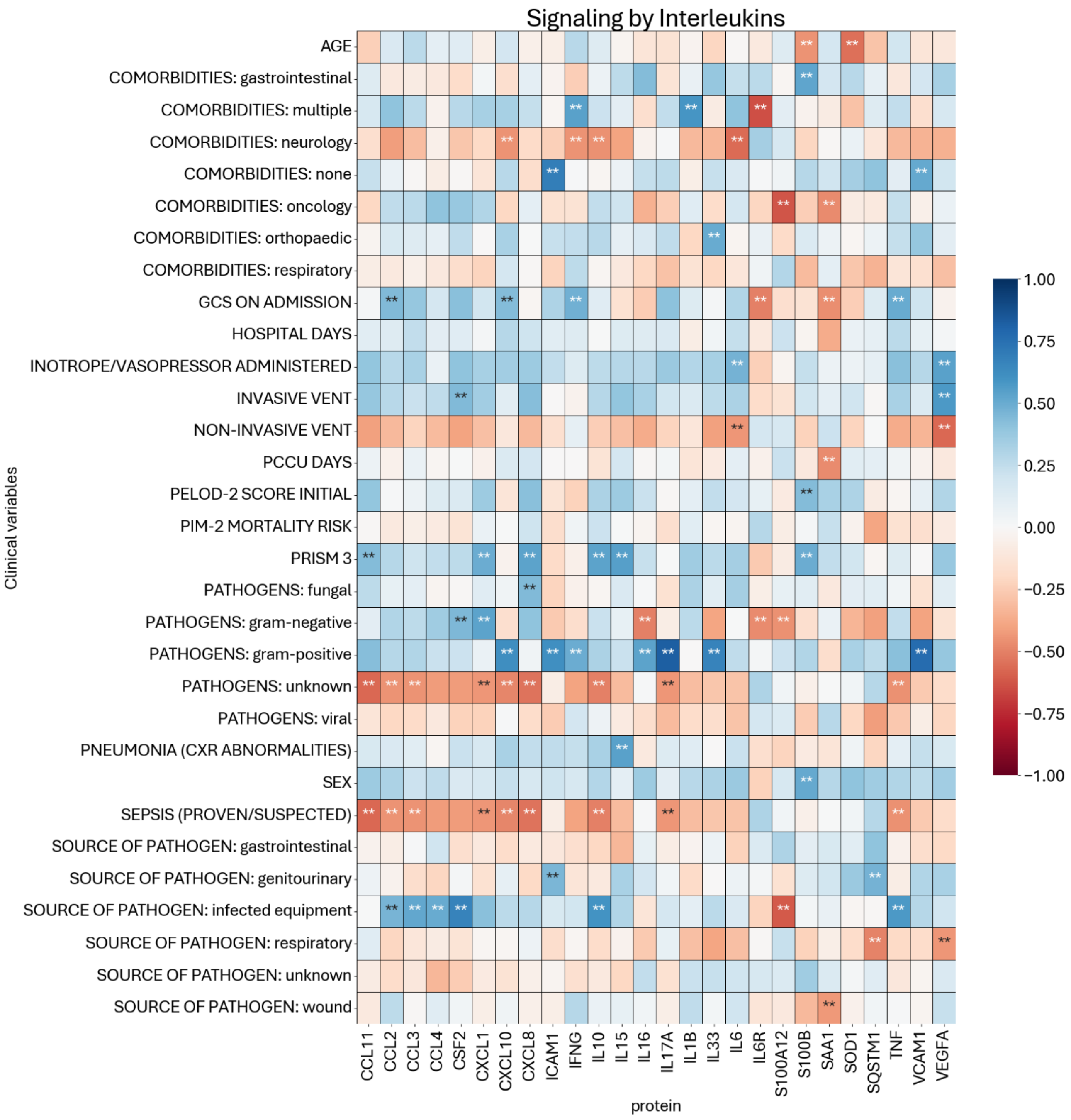

Supplementary Figure 5.

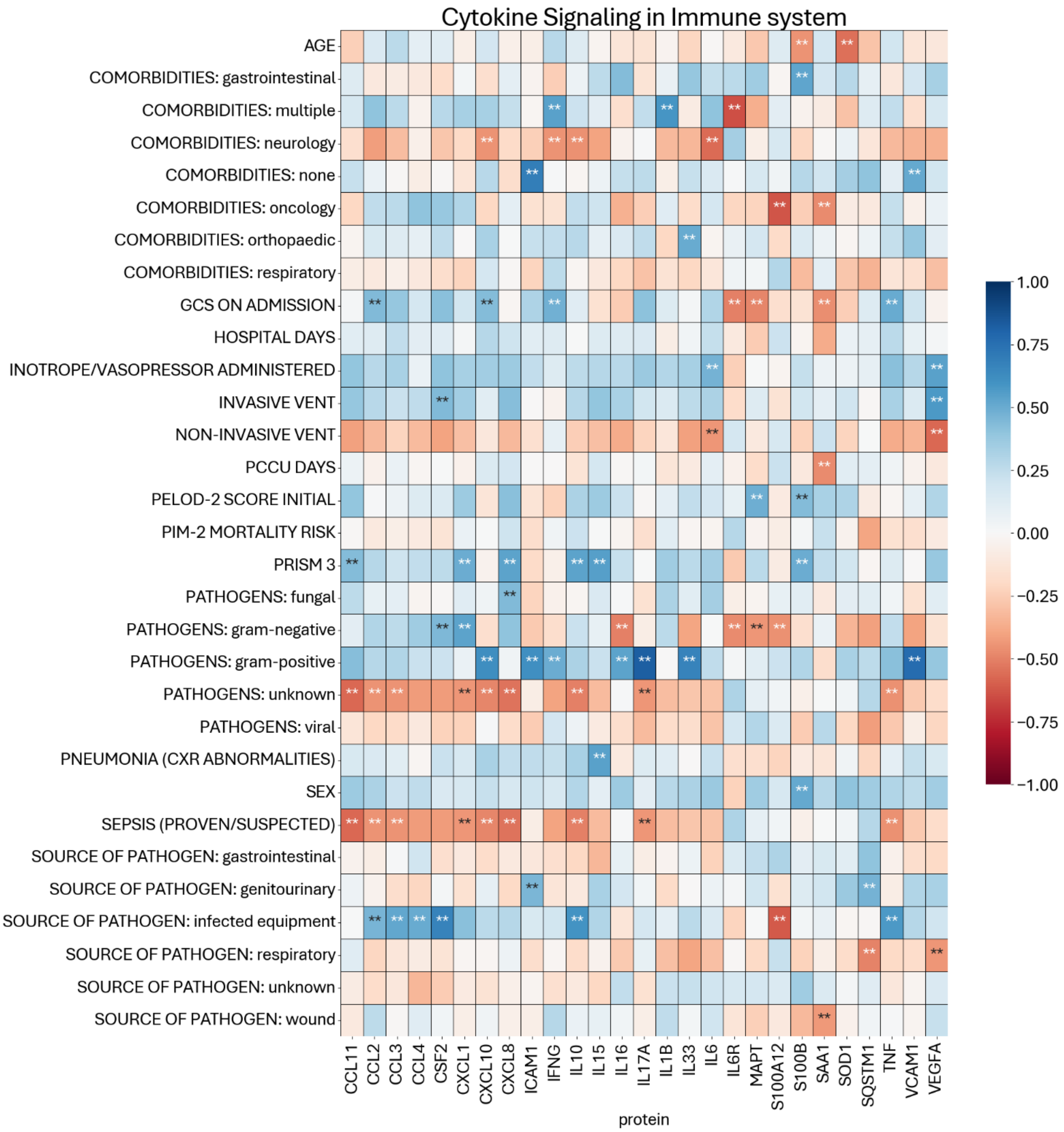

Supplementary Figure 6.

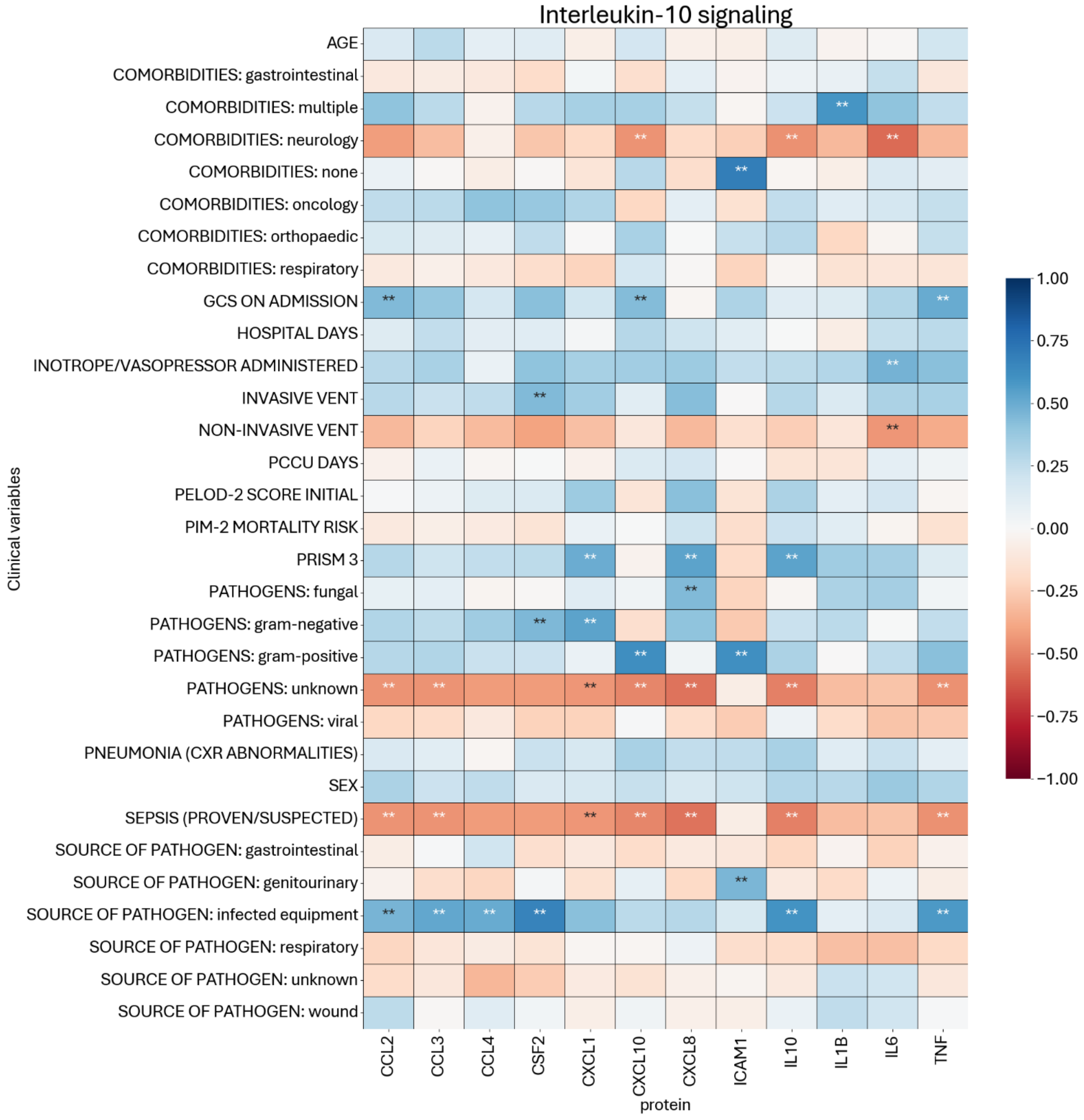

Supplementary Figure 7.

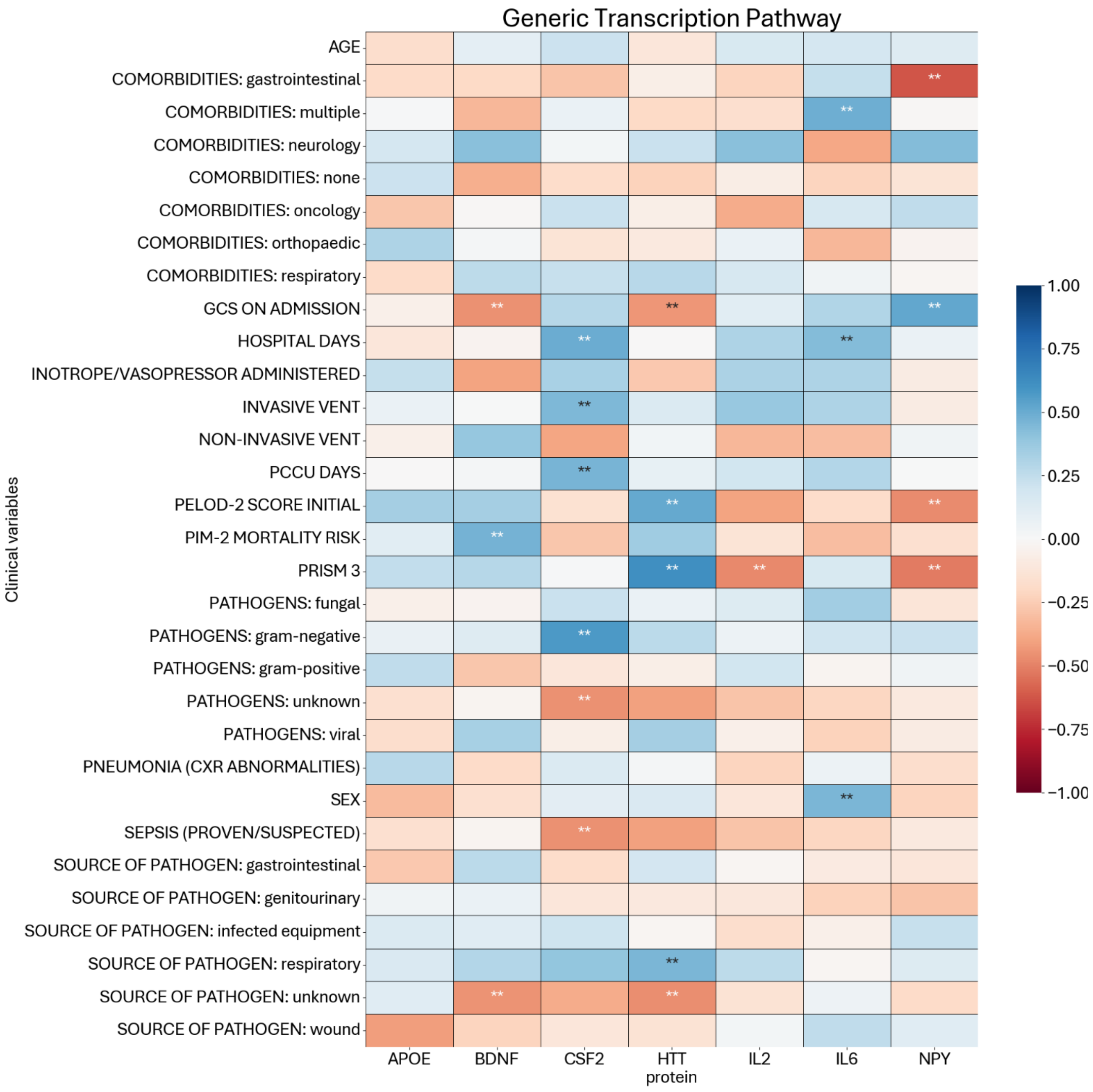

Supplementary Figure 8.

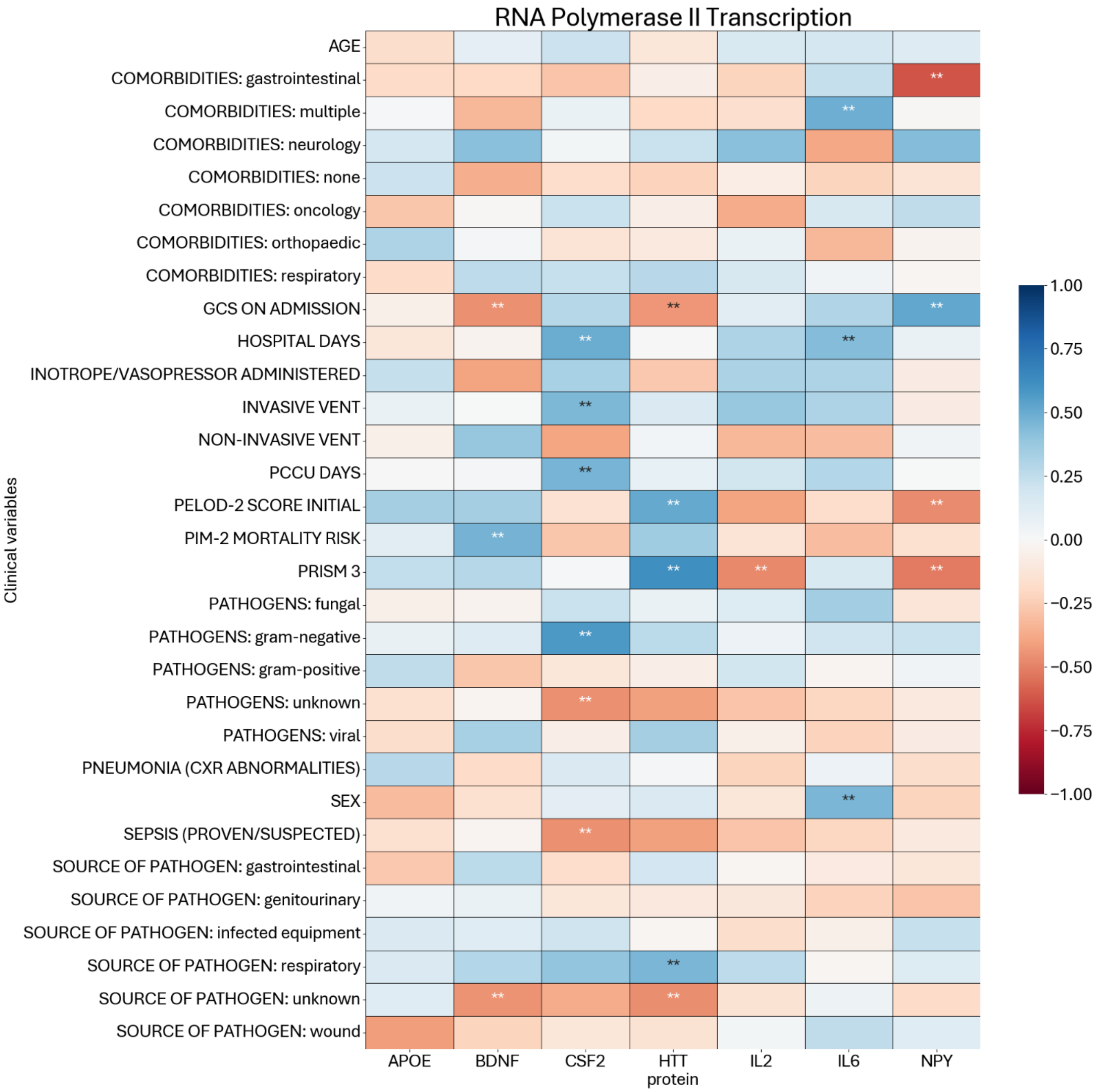

Supplementary Figure 9.

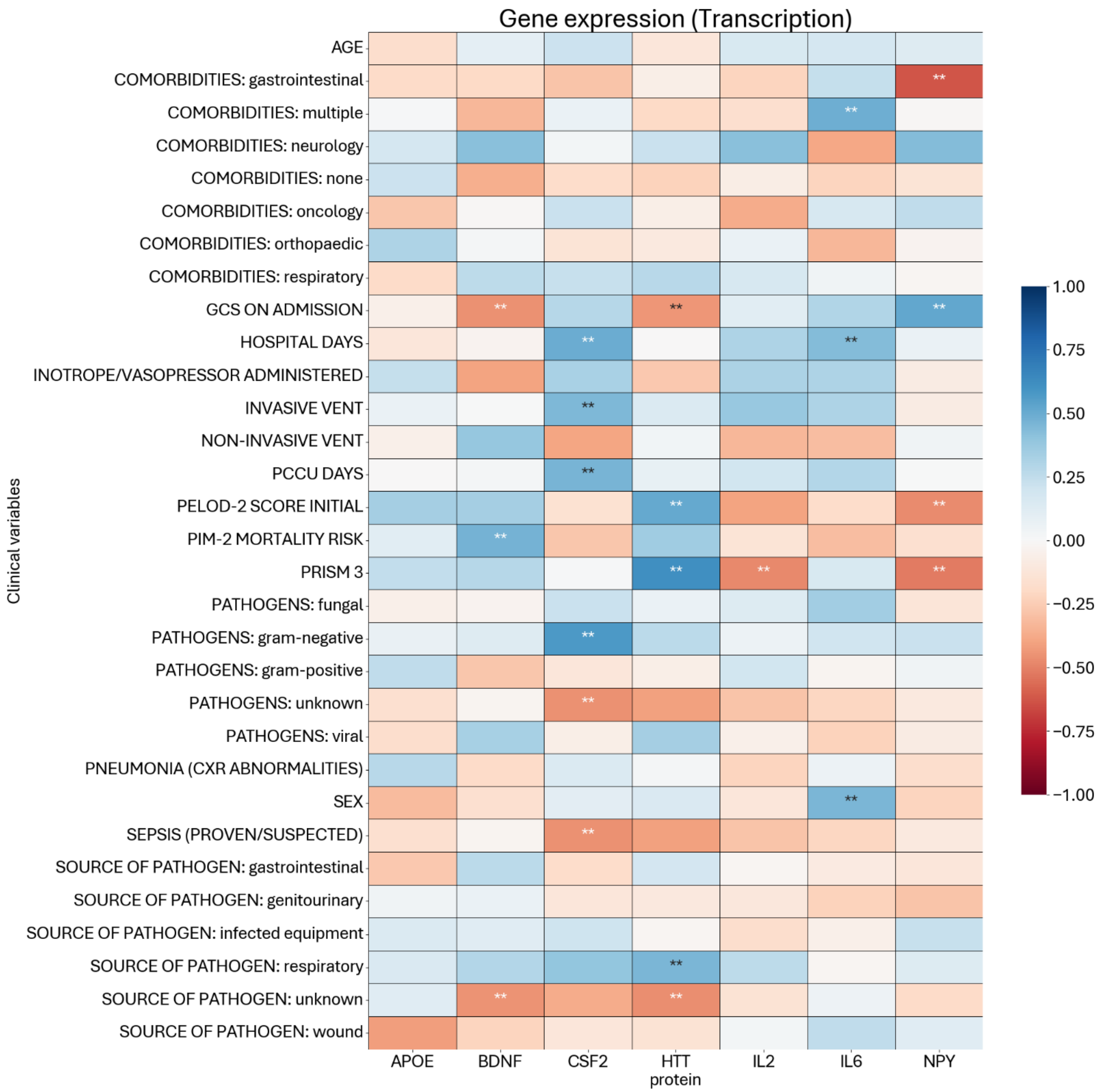

Supplement: Supplementary file 1 — Supplementary Material 1. [file 10020_2025_1397_MOESM1_ESM.pdf]
